# Role of decomposition products in the oxidation of cyclohexene using a manganese(III) complex

**DOI:** 10.1038/s42004-023-00881-x

**Published:** 2023-05-17

**Authors:** Zahra Zand, Younes Mousazade, Ryan Lacdao Arevalo, Robabeh Bagheri, Mohammad Reza Mohammadi, Rahman Bikas, Petko Chernev, Pavlo Aleshkevych, Matthias Vandichel, Zhenlun Song, Holger Dau, Mohammad Mahdi Najafpour

**Affiliations:** 1 Department of Chemistry, Institute for Advanced Studies in Basic Sciences (IASBS), Zanjan, 45137-66731 Iran; 2 Department of Chemical Sciences and Bernal Institute, University of Limerick, Limerick, V94 T9PX Ireland; 3 Key Laboratory of Marine Materials and Related Technologies, Zhejiang Key Laboratory of Marine Materials and Protective Technologies, Ningbo Institute of Materials Technology and Engineering, Chinese Academy of Sciences, 315201 Ningbo, China; 4 University of Sistan and Baluchestan, Department of Physics, Zahedan, 98167-45845 Iran; 5 Department of Chemistry, Faculty of Science, Imam Khomeini International University, 34148-96818 Qazvin, Iran; 6 Fachbereich Physik, Freie Universit-t Berlin, Arnimallee 14, 14195 Berlin, Germany; 7 Department of Chemistry, Ångström Laboratory, Uppsala University, Box 523, Uppsala, 751 20 Sweden; 8 Institute of Physics, Polish Academy of Sciences (PAN), Al. Lotnikow 32/46, PL-02-668 Warsaw, Poland; 9 Center of Climate Change and Global Warming, Institute for Advanced Studies in Basic Sciences (IASBS), Zanjan, Iran; 10 Research Center for Basic Sciences & Modern Technologies (RBST), Institute for Advanced Studies in Basic Sciences (IASBS), 45137-66731 Zanjan, Iran

**Keywords:** Heterogeneous catalysis, Organometallic chemistry

## Abstract

Metal complexes are extensively explored as catalysts for oxidation reactions; molecular-based mechanisms are usually proposed for such reactions. However, the roles of the decomposition products of these materials in the catalytic process have yet to be considered for these reactions. Herein, the cyclohexene oxidation in the presence of manganese(III) 5,10,15,20-tetra(4-pyridyl)-21H,23H-porphine chloride tetrakis(methochloride) (**1**) in a heterogeneous system via loading the complex on an SBA-15 substrate is performed as a study case. A molecular-based mechanism is usually suggested for such a metal complex. Herein, **1** was selected and investigated under the oxidation reaction by iodosylbenzene or (diacetoxyiodo)benzene (PhI(OAc)_2_). In addition to **1**, at least one of the decomposition products of **1** formed during the oxidation reaction could be considered a candidate to catalyze the reaction. First-principles calculations show that Mn dissolution is energetically feasible in the presence of iodosylbenzene and trace amounts of water.

## Abstract (web summary)

Metal complexes are often used as catalysts for oxidation reactions, however, there are open questions about the role of the decomposition products in the catalytic process. Here, the authors explore the potential role of decomposition products in the oxidation of cyclohexene using a manganese(III) complex catalyst adsorbed on an SBA-15 substrate.

## Introduction

The selective oxidation of various chemicals into beneficial products is necessary for laboratory and synthetic industrial applications^1–3^. Metal complexes are extensively used as the catalysts for these reactions; molecular-based mechanisms are usually proposed for such reactions^4,5^. However, in contrast to water oxidation, water reduction, and hydrogenation reactions^6,7^, the roles of the decomposition products of the catalyst as one of the candidates for active transformed species (true catalyst) have yet to be deeply investigated for other reactions. Recognizing the true catalyst is critical for finding the mechanism, calculating turnover frequency, and optimizing the reaction. It is suggested that the decomposed molecular structure could produce species that serve as the catalysts for the related reaction because:

(i) The harsh conditions of many organic oxidation reactions and the use of powerful oxidants such as oxone, peroxides, and iodosylbenzene could decompose the molecular structure of (pre)catalyst.
(ii) Using relatively high temperatures in some oxidation reactions induces the decomposition reaction^8^.
(iii) In some oxidation reactions, the highly pure metal complexes could not be easily obtained, and the presence of an impurity in the form of the metal salt is inevitable.
(iv) The catalytic activity was usually calculated after a long time, rendering the decomposition of the metal complex inevitable^9–14^.
(v) Some metal complexes need the activation time for the oxidation reaction. “Why this activation time is necessary” is an enigma in the context of oxidation reactions.
(vi) In the same oxidation reaction conditions, which occurs in the presence of metal complexes, other simple metal compounds such as metal salts^15,16^, metal oxide and^17–20^ uncomplexed metal ions on different supports^21,22^ are active toward the attributed oxidation reaction.

Numerous metal complexes with different ligands have been investigated for the oxidation of different organic compounds^23–30^. Herein, manganese(III) 5,10,15,20-tetra(4-pyridyl)-21H,23H-porphine chloride tetrakis(methochloride) (**1**) is used as an interesting catalyst for the cyclohexene oxidation. It is proposed that at least one produced compound during the reaction other than the metal complex can be a candidate for the true catalyst.

Finding the oxidation mechanism and the true catalyst for the related reaction is necessary to design and synthesize efficient catalysts for oxidation reactions. A molecular-based mechanism is usually suggested for the complexes such as **1**^5^.

Herein, **1** supported on Santa Barbara Amorphous material (SBA-15) substrate is selected and investigated under the oxidation reaction. It is proposed that, in addition to **1**, at least one of the decomposition products of **1** formed during the oxidation reaction could be considered as a candidate to catalyze the oxidation reaction.

## Results and discussion

**1** was immobilized on SBA-15 (SBA) by electrostatic interaction using a previously reported method^29^. The immobilized **1** on silica SBA (SBA-**1**) as a catalyst was investigated under cyclohexene oxidation using iodosylbenzene (PhIO) as an oxidant reagent. With an oxidant: catalyst ratio of 10:1 after 30 min (SBA-**1030**) and 90 min (SBA-**1090**) and with an oxidant: catalyst ratio of 100:1, keeping the catalyst concentration constant after 30 min (SBA-**10030**) and 90 min (SBA-**10090**), the solid was separated, washed and investigated^29^.

XRD showed that **1** is amorphous. The broad diffraction peak at 20–30° was observed in all compounds containing SBA related to amorphous silica. The presence of **1** could not be detected in the samples because of its amorphous structure (Fig. S1).

**Fig. 1:**
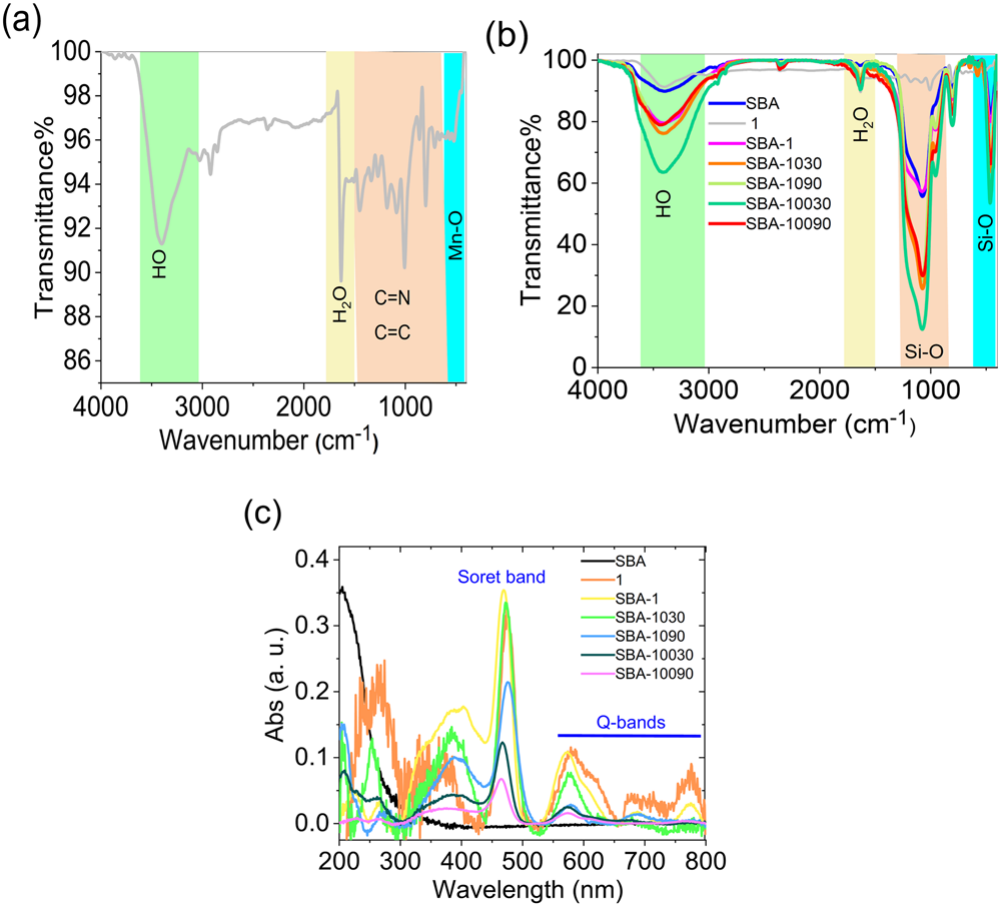
FTIR spectrum of **1** (**a**); FTIR spectra of SBA, **1**, SBA-**1**, SBA-**1030**, SBA-**1090**, SBA-**10030**, and SBA-**10090** (**b**); Diffuse reflectance UV–Vis spectra of SBA, **1**, SBA-**1**, SBA-**1030**, SBA-**1090**, SBA-**10030**, and SBA-**10090** (**c**). UV–Vis spectra of SBA-**1**/PhIO/cyclohexene molar ratio of 1:100:5000, MeCN: CHCl_3_ (1:1, v/v) as a solvent, magnetic stirring, 25 °C, at room atmosphere (air), reaction time: 120 min.

The FTIR spectra were recorded using KBr plates in 500–4000 cm^−1^. FTIR spectrum of **1** shows the peaks at 750–1580 cm^-1^ correspond to the C = C and C = N vibrations of the ligand (Fig. 1a). FTIR spectra of the samples containing SBA are similar (Fig. 1b). They show a broad band at ~3200-3600 cm^−1^ related to the antisymmetric and symmetric O-H stretching modes (Fig. 1b). The H-O-H bending at ~1630 cm^−1^ is also observed (Fig. 1b). The Si–O stretching peak is observed at 1040–1100 cm^−1^. The stretching vibration of Si–O–Si is observed at 1030 cm^−1^ (Fig. 1b)^30^. Due to the small amount of **1** on SBA, it is impossible to detect **1** in these materials using many routine methods such as FTIR, elemental analysis, and thermogravimetry.

All these materials were characterized by diffuse reflectance UV–Vis spectroscopy. For samples containing **1**, the Soret band^15^ is observed at 470 nm (Fig. 1c). Q-bands^15^ are also observed at 550–800 nm (Fig. 1c). The Soret band and Q-bands for **1** and the immobilized **1** are similar (Fig. 1c). The color of **1** in this heterogeneous catalyst disappeared slowly during the reaction. Thus, the immobilized **1** on SBA is decomposed during the reaction; it is hypothesized that the decomposed compound(s) has no clear UV–Vis spectra and, therefore could not be detected easily by UV–Vis spectroscopy. A change in 300–400 nm during the reaction was observed. In addition, Mn(II) and small fragments of the ligand, if formed during the reaction, could not be easily detected by UV–Vis spectroscopy. The color of a solution of **1** in the presence of PhIO disappeared. Thus, color disappearance is not related to the leaching out from the SBA support but the decomposition of **1**.

SBA-**10030**, among other samples, was selected and investigated by XPS. The XPS shows C, O, and I on the surface of SBA-**10030** (Fig. S2a). In addition to the peak for C at 284.8 eV related to C–C, two peaks at 286.1 and 287.9 eV are observed related to the oxidized carbon such as C–O/C–N (Fig. S2b). The peaks at 530.4 eV, 531.8 eV, 533.5 eV, and 534.7 eV are related to Si–O, C–O, H_2_O, and C = O (Fig. S2c), respectively^31^. I 3d_5/2_ and I 3d_3/2_ are observed at 623.33 eV and 635.2 eV (Δ = 11.87 eV), respectively. I 3d_5/2_ for iodide ion in metal iodides is observed at 619 eV. For iodide, the I 3d region has well-separated spin-orbit components (Δ = 11.5 eV). However, I 3d_5/2_ at 623.33 eV is higher than the energy of I 3d_5/2_ iodide. Such high energy for I 3d_5/2_ is related to I–O compounds and corresponds to iodocylbenzene^32^. Given the small amount of Mn on the surface of SBA, no Mn is detected by XPS. It is suggested that large amounts of Mn is released into the solution and no Mn could be detected by XPS.

Unsurprisingly, the SEM images (for SEM images, see Figs. S3–S14) of all these compounds show relatively similar morphology to SBA, which shows no significant change after the immobilization of **1**. The SEM images indicate the presence of elongated, 200–300 nm wide vermicular-shaped particles with 2–3 μm lengths. SEM-EDX shows that in a long-time oxidation reaction, manganese could be observed in the solid after the decomposition of 1 and the elimination of the color of compounds. The SEM-EDX displays C, O, Si, and I on the surface of SBA-**1030**, SBA-**1090**, SBA-**10030**, and SBA-**10090** (Figs. S3–S14). On the surface of SBA and SBA-**1**, only O, C, and Si are detected. All elements are dispersed on the surface of solids.

(High-resolution) transmission electron microscopy ((HR)TEM) images of SBA-**1090**, SBA-**10030**, and SBA-**10090** are shown in Figs. S15–S19. SBA-15 has a mesoporous structure and a small number of micropores and indicates hexagonal pores in a 2D array with long 1D channels (*p*6*mm* plane group). The same structure could be observed for SBA-**1090**, SBA-**10030**, and SBA-**10090**^33^. Such channels are interconnected by small micropores and can mitigate the diffusion barrier between the reactants and the products.

Mn oxides as a separated phase are not detected by SEM or TEM for SBA-**1090**, SBA-**10030**, and SBA-**10090** using SEM or TEM. Thus, the Mn compounds on the surface of SBA could be **1** or (and) other Mn compounds than Mn oxide.

**Fig. 2:**
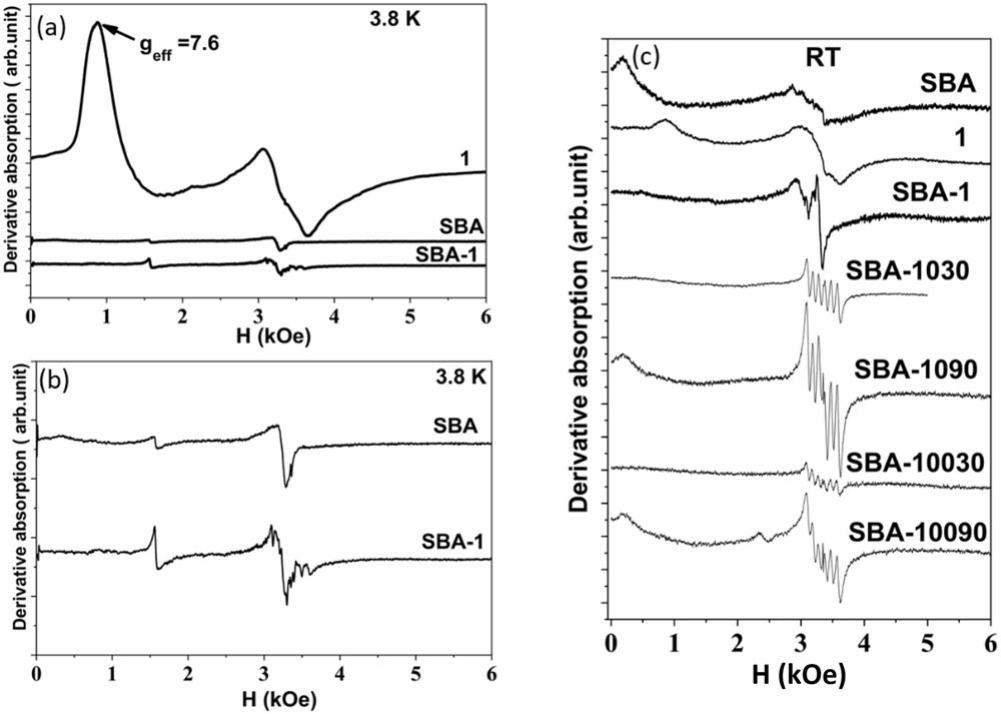
EPR spectra for powdered **1**, SBA, and SBA-**1** recorded at liquid helium temperature (**a**, **b**). EPR spectra of SBA, **1**, SBA-**1**, SBA-**1030**, SBA-**1090**, SBA-**10030**, and SBA-**10090** for powdered complexes recorded at room temperature (**c**).

The compounds are further studied by EPR spectroscopy. Since the samples were powders, the paramagnetic centers were randomly orientated concerning the magnetic field, and the spectra represented the envelope of all resonance lines summed over all possible orientations. The spectrum of SBA, used as a reference spectrum, shows negligible resonance absorption from residual paramagnetic impurities (Fig. 2a). At low temperatures, a characteristic peak near H = 1620 (Oe) becomes visible in the SBA spectrum and is typically attributed to Fe(III) impurities in a strong axially distorted environment.

The EPR spectrum of **1** shows two resonances at low temperatures (Fig. 2a); the low peak is characterized by an effective g-factor of ≈ 7.6, and the unresolved structure is centered around g = 2. The low peak is associated with Mn(III) (d^4^, S = 2) ions. In general, the X-band EPR spectroscopy is not applicable for probing integer-spin magnetic ions. However, Mn(III), in some particular coordination, allows observation of the resonance attributed to usually forbidden transitions within the |±2> non-Kramers doublet^34^. In the spectrum of Mn porphyrin on SBA (SBA-**1**), only a small amount of absorption, unrelated to Mn ions, is possibly due to the presence of a trace amount of iron in SBA is observed (Fig. 2b).

As shown in Fig. 2c, at room temperature, the EPR spectra of SBA-**1030**, SBA-**1090**, SBA-**10030**, and SBA-**10090** exhibit intensive sextet centered at g = 2.0. This sextet is a characteristic of Mn(II) ions and results from the hyperfine splitting structure due to nuclear spin I = 5/2 of ^55^Mn ion within the Kramers spin doublet |±1/2 > . The hyperfine splitting in SBA-**1030**, SBA-**1090**, SBA-**10030**, and SBA-**10090** is identical and characterized by the same splitting constant *A* = 0.009(0) cm^-1^ (270 MHz). The obtained value of *A* is close to a six-coordinated Mn(II) in [Mn(H_2_O)_6_]^2+^: 0.0089 cm^−1,^^35^. The oxidation operation performed by PhI(OAc)_2_ as a comparative oxidant led to the same EPR pattern for SBA-**1** after operation using oxidant: catalyst ratio of 10:1 (SBA-**1024AC**) and 100:1 (SBA-**10024AC**) after 24 h (Fig. S20).

**Fig. 3:**
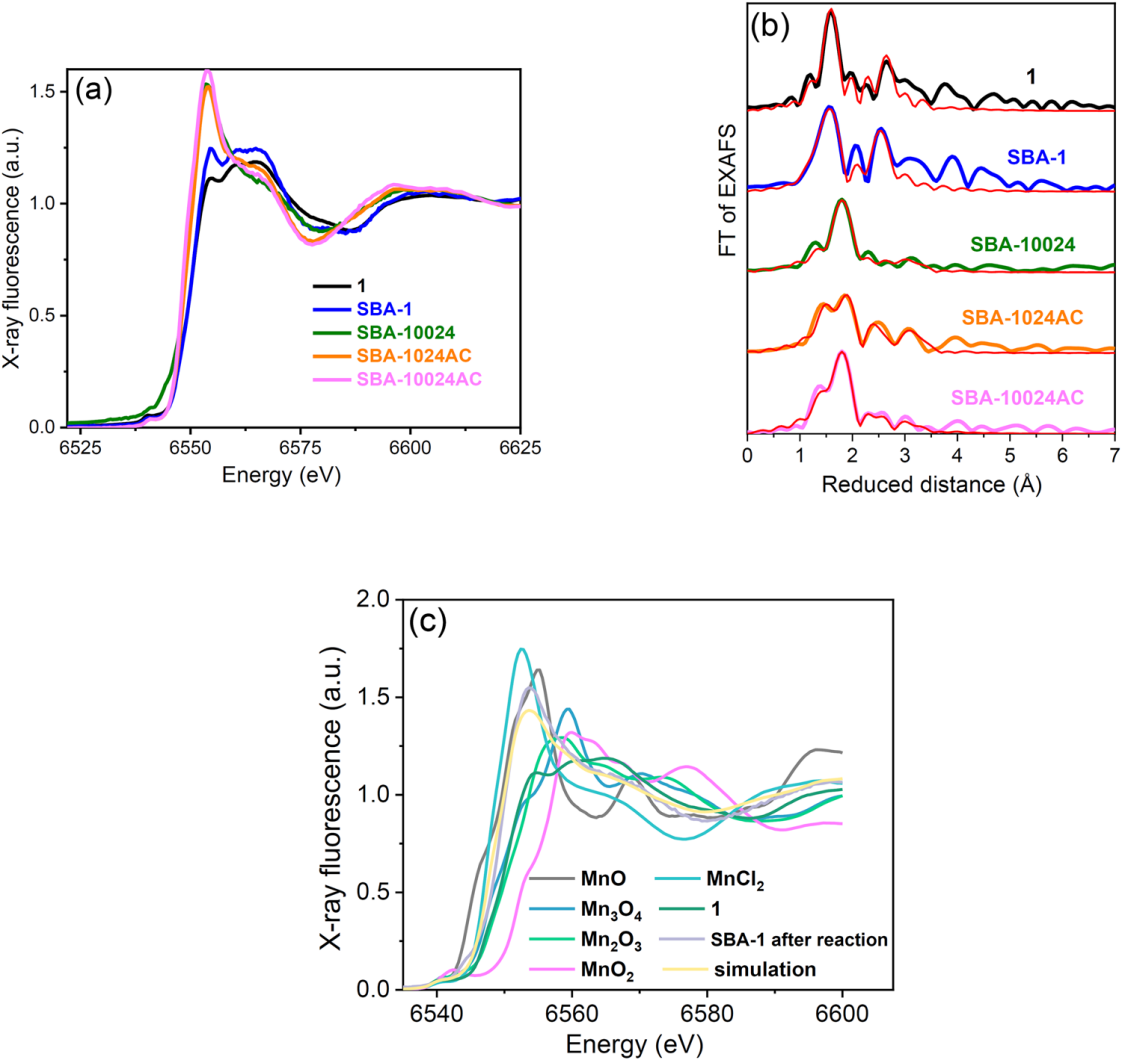
XANES spectra (**a**) and Fourier-transform of the k^3^-weighted EXAFS spectra (**b**) of **1**; SBA-**1**, SBA-**10024**, SBA-**1024AC**, and SBA-**10024AC**. The thick lines show experimental data, and the thin red lines show simulations. The phase shift is not corrected. The k^3^-weighted EXAFS oscillations are shown in Fig. S21. The fit parameters for the simulations are given in Table 1. Linear combination of different reference oxide edges and the edge of the original complex (**1**) to fit the edge of the SBA-**10024** sample (**c**).

In the next step, we used X-ray absorption spectroscopy (XAS) to obtain information on the decomposition products of the catalyst after 24 h catalytic operation using two oxidants: PhIO (with the oxidant: catalyst ratio of 100:1 (SBA-**10024**)) and PhI(OAc)_2_ (with the oxidant: catalyst ratio of 100:1 and 10:1 (sample SBA-**10024AC** and SBA-**1024AC**, respectively)). Different oxidants were used to follow the true catalyst formation phenomenon and generalize the concept. Figure 3a shows the Mn K-edge, where the edge positions of **1** and SBA-**1** are approximately identical. After reaction (24 h), for SBA-**10024**, the edge position shifts from 6549.5 to 6547.9 eV, corresponding to the average Mn oxidation states of 2.9 and 2.4, respectively, as calculated by comparison to edge positions of Mn compounds with known oxidation states (Fig. S22). XANES also shows that the original complex was decomposed because the shape of the edge was completely changed. The Fourier transform of the experimental EXAFS spectrum of **1** can be readily reproduced by a simulation that directly uses its molecular structure (Fig. 3b). The spectrum shows two main peaks, where the first high main peak can be simulated by two Mn–N/Cl shells at 1.94 Å and 1.95 Å for the original compound (**1**) and 1.86, 2.00 and 2.05 Å for the SBA-**1** (Fig. 3b, and Table 1). After the oxidation reaction, all three measured samples showed EXAFS that differ from **1**. The first main peak shifted to a higher distance, and the second peak disappeared, suggesting a rearrangement of the structure that K-edge XANES already confirmed. The three samples showed slightly different EXAFS, presumably due to the different ratios of decomposition products achieved in the other conditions. For SBA-**10024**, for example, a linear combination of different reference-oxide edges and the edge of the original complex to fit the edge of the material after the reaction indicates the presence of Mn(II) hexa aquo (55%), no amount of the original complex (**1**), and Mn_2_O_3_ (45%) (Fig. 3c). The EXAFS after the reaction for all three samples can be simulated as a mixture of long and short Mn-O distances, again suggesting a mixture of Mn(II) and Mn(III) coordinated to O.

**Table 1:** Parameters obtained by simulating the k^3^-weighted EXAFS spectra of Mn K-edge of **1**, SBA-**1**, SBA-**10024**, SBA-**1024AC**, and SBA-**10024AC**.

Type of bond	1		SBA-1	
	R [Å]	N	R [Å]	N
**Mn–N**	1.95 (0.01)	4	1.86 (0.03)	1
**Mn–N**	–	–	2.00 (0.01)	3
**Mn–Cl**	1.94 (0.04)	1	2.05 (0.03)	1
**Mn–C**	2.81 (0.06)	1.1	–	–
**Mn–C**	3.04 (0.01)	6.9 (0.9)	3.01 (0.4)	8

Type of bond	SBA-10024		SBA-1024AC		SBA-10024AC	
	R [Å]	N	R [Å]	N	R [Å]	N
**Mn–O**	1.89 (0.07)	0.7 (0.5)	2.01 (0.02)	2.3 (0.5)	1.98 (0.05)	1.6 (0.6)
**Mn–O**	2.12 (0.07)	3.1 (2.3)	2.20 (0.02)	4.2 (0.6)	2.18 (0.03)	5.5 (0.8)
**Mn–O**	2.24 (0.09)	2.1 (2.0)	2.91 (0.03)	2.5 (1.2)	2.35 (0.09)	1.3 (0.9)

The simulated spectra correspond to the Fourier-transformed EXAFS spectra shown in Fig. 3b. To avoid over-parametrization, the Debye-Waller parameters (σ) were set to 0.049 Å, 0.093 Å, 0.049 Å, and 0.067 Å for nitrogen, chloride, carbon, and oxygen, respectively. The simulation program determined the coordination number (N) and backscattering distance (R). The amplitude reduction factor, S_0_^2^, was fixed at 0.7. The filtered R-factors were 25, 21, 16, 25, and 15 for the original compound (**1**), SBA-**1**, SBA-**10024**, SBA-**1024AC**, and SBA-**10024AC**, respectively. The reduced chi-squared values for Mn complex (**1**), SBA-**1**, SBA-**10024**, SBA-**1024AC**, and SBA-**10024AC** were 29, 85, 13, 13, and 15, respectively. Errors are represented in parentheses.

First-principles calculations based on density functional theory (DFT) were performed to shed light on the decomposition of **1** in the presence of PhIO within the B3LYP functional. The structures were optimized at the B3LYP/6-31 G* level, followed by single-point energy refinement employing the B3LYP/Def2TZVP with implicit MeCN solvent (dielectric constant of 37.5) via the PCM approach^36^. All calculations were carried out using the Gaussian16 program^37^. Table 2 shows the relative energies of **1** for different spin multiplicities, referenced to the singlet state, and the average Mn–N bond lengths. It can be noted that **1** prefers a quintet multiplicity state, which has the longest Mn–N bond length compared to the other multiplicities. The longer Mn–N bond lengths for the high spin states are consistent with an earlier noted elongation of the Mn–ligand distances for spin-crossover from the low to high spin states of Mn complexes^38,39^.

**Table 2:** Energies of **1** and Mn–N Bond Lengths for different spin multiplicities.

Spin Multiplicities	Energy (eV)	Mn–N Bond Length (Å)
Singlet	0.000	1.972
Triplet	–1.465	1.996
Quintet	–2.655	2.017
Septet	–1.095	2.014

**Fig. 4:**
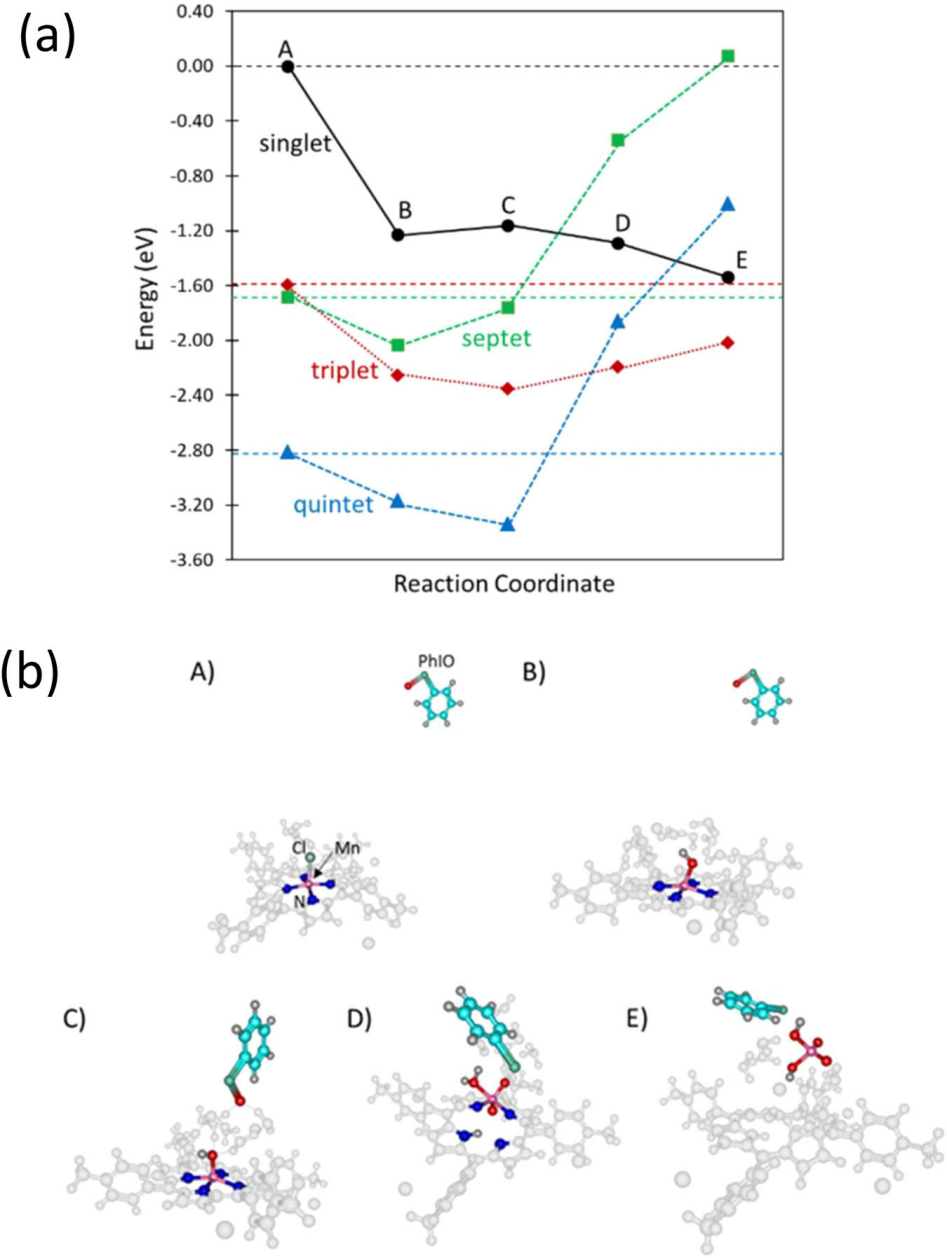
The relative energies of the explored decomposition pathway of **1** for different spin multiplicities (**a**) and the optimized structures of a system for the triplet state as a representative structure (**b**).

It can be inferred from EXAFS results that showed the presence of Mn atoms coordinated with O after a 24 h reaction, the possible dissolution of Mn. Such a pathway leading to the release of Mn into the solution was modeled with **1** initially solvated by a cluster of eight water molecules, followed by a PhIO attack on the Mn–N bond and Mn dissolution. Figure 4 shows the relative energies of the different states comprising the explored pathway for the decomposition of **1** with different spin multiplicities. At point A, water clusters surround the complex centre with the PhIO far from the system. At point B, the Mn atom is oxidized. The reaction from A to B proceeds exothermically for all the spin multiplicities. At point C, PhIO adsorbs on the water cluster forming H-bonds with water molecules. This process is exothermic for the triplet and quintet states and slightly endothermic for the other spin multiplicities. At point D, PhIO attacks the Mn–N bond, forming a MnO_2_^.^H_2_O moiety from the dissociated O atom from PhIO. It can be noted from Fig. 4 that such a process is exothermic for the singlet state but endothermic for the other spin multiplicities. Finally, at point E, Mn is released into the solution forming a MnO_2_(OH)_2_ molecule. This step proceeds downhill for the singlet state and uphill for the other spin multiplicities. Notably, point E is most stable in the triplet state. The formed high valence manganate at point E is unstable in the presence of organic solvent and may react with the ligand of **1**^40^. Such instability of manganate is illustrated in a previous study where KMnO_4_ is easily transformed into K_3_(MnO_4_)_2_, δ-MnO_2_, and O_2_^41^. The facile release of Mn in the solution supports the earlier noted absence of Mn compounds on the surface of SBA as observed from SEM and TEM results, as well as the presence of Mn atoms coordinated with O after a 24 h reaction as suggested by the earlier mentioned EXAFS results. The energy landscape in Fig. 4 shows that the explored reaction pathway proceeds downhill in energy for the singlet state. For the triplet state, the most endothermic step is from point D to E, with an energy difference of only 0.178 eV. This indicates that the explored decomposition pathway can also proceed easily in the triplet state. For the quintet state, while path A → B → C proceeds downhill in energy, the C to D step requires an energy of 1.48 eV. Meanwhile, points D and E are more stable for the triplet state than for the quintet state. This implies a possibility of a spin-crossover from point C in the quintet to point D in the triplet state.

In the next step, the effect of SBA, SBA-**1**, and SBA/manganese(II) acetate (MnAc) on the oxidation of cyclohexene in the presence of an oxidant, iodosylbenzene, was investigated (Eq. 1). The reaction products were monitored by gas chromatography using bromobenzene as an internal standard.

At the relatively low oxidant ratio to the catalyst (10:1) for SBA-**1** (entry 1 in Table 3), 15% of epoxide and 20% of ketone were observed after 90 min. Cyclohexene (62%) was also detected without any reaction. For SBA/MnAc (entry 2), 7% of epoxide and 8% of ketone were obtained after 90 min. At the high ratio of oxidant to catalyst (100:1) for SBA-**1** (entry 3), 11% of epoxide and 10% of ketone were detected after 90 min. For SBA/MnAc (entry 4), 12% of epoxide and 8% of ketone were observed after 90 min. One hypothesis is that **1** in SBA-**1** could be decomposed to simple Mn salt. At the high ratio of oxidant to catalyst (100:1) for SBA-**1** (entry 5), after 24 h, a small amount of ketone (8%) and larger amounts of epoxide (22%) were observed. For SBA/MnAc (entry 6), after 24 h, 12% of epoxide and 20% of ketone were observed. Interestingly, after the reaction of SBA-**1** and the oxidant (1:100) for 24 h, the substrate and the oxidant (reused catalyst: fresh oxidant, 1:10) were added, and the results showed 15% of epoxide and 20% of ketone (entry 7). SBA without **1** showed only ketone (8%) after 24 h (oxidant: SBA = 10:1). This activity of SBA may be related to iron impurity, as was detected by EPR spectroscopy.

**Table 3:** The oxidation of cyclohexene in the presence of iodosylbenzene under different conditions.


Entry	Catalyst	PhIO: Catalyst	Time	Yield^a^
1	SBA-**1**	10:1	90 min	1: 15% 2: 20%
2	SBA/MnAc	10:1	90 min	1: 7% 2: 8%
3	SBA-**1**	100:1	90 min	1: 11% 2: 10%
4	SBA/MnAc	100:1	90 min	1: 12% 2: 8%
5	SBA-**1**	100:1	24 h	1: 22% 2: 8 %
6	SBA/MnAc	100:1	24 h	1: 12% 2: 20%
7^b^	Reused SBA-**1**	10:1	90 min	1: 15% 2: 20%
8	SBA-**1**	100:1	48 h	1: 30% 2: 15%
9	SBA/MnAc	100:1	48 h	1: 15% 2: 25%
10	PhIO	10:0	24 h	2: 5%
11	SBA	10:1	24 h	2: 8%

^a^The yield products were monitored by gas chromatography using bromobenzene as an internal standard.

^b^The reused SBA-**1** catalyst was recovered from the reaction of entry 5.

For more investigation, at the high ratio of catalyst to oxidant (1:100) for SBA-**1** after 48 h (entry 8), 30% of epoxide and 15% of ketone were detected. For SBA/MnAc (entry 9), the amounts of ketone (25%) and epoxide (15%) were observed after 48 h. All these experiments showed that:

1. SBA/MnAc has activity toward the oxidation of cyclohexene.
2. At least under some conditions, SBA/MnAc shows the activity of SBA-**1** toward the oxidation of cyclohexene.
3. The low catalytic yield was observed when more oxidant was used (catalyst: oxidant = 1:100). However, when this catalyst was recovered and reused in the second cycle of reactions at a lower molar ratio (catalyst:oxidant=1:10), the catalytic yields significantly improved. It is possible that the catalyst in the first stage, which took place under harsh conditions (catalyst:oxidant=1:100), decomposed and formed new catalysts for the second cycle.

The oxidation of cyclohexene using SBA-**1** was also investigated under the same conditions by three other oxidants: (Diacetoxyiodo)benzene (PhI(OAc)_2_), meta choloroperbenzoic acid (MCPBA), and oxone tetrabutylammonium (TBA-OX). The results of the reactions are represented in Tables (S1, S2, and S3).

The oxidation reaction by PhI(OAc)_2_ in the presence of SBA-**1** or SBA/MnAc showed the formation of an epoxide product. SBA-**1** was separated and washed after 24 h catalytic activity, then used again in the same condition. Reused SBA-**1** shows some activity in the second run (Table S1). On the other hand, the results of reactions using MCPBA and TBA-OX show that neither the SBA-1 nor SBA/MnAc are active in the conditions (Tables S2, 3).

### The multi-catalyst formation during the oxidation reaction could address and solve some of the ambiguities in the field:^42–44^

1. Selectivity and turnover numbers for oxidation reactions in the presence of the metal complexes are different at different reaction times^10,45,46^. The change in catalysts during the reaction may cause such an effect.
2. In the required activation time for the metal complexes in the presence of oxidants, the precatalyst could be decomposed, and a true catalyst is formed.
3. The oxidation reaction in the presence of powerful oxidants, especially at higher temperatures than room temperature^45^ and over a long time^9,14^, could decompose the metal complexes.
4. In some cases, metal complexes with lower stability show higher activity for an oxidation reaction^47^. In this case, the large amounts of decomposed complexes form the more significant amounts of catalysts.

## Conclusions

The recognition of the true catalysts was rarely investigated in the oxidation of organic compounds. Manganese(III) 5,10,15,20-tetra(4-pyridyl)-21H,23H-porphine chloride tetrakis(methochloride) (**1**) was used as a (pre)catalyst for the cyclohexene oxidation as a study case. Some methods showed that **1** was not stable and decomposed to the simple manganese(II) compounds under the oxidation conditions in the presence of iodosylbenzene. This is supported by the relatively facile dissolution of Mn as determined by first-principle calculations. Simple manganese salts such as manganese acetate are catalysts for cyclohexene oxidation; thus, in addition to **1**, it is proposed that at least one of the decomposition products of **1** could also be considered as one of the candidates for the true catalyst for the oxidation reaction, especially during a long-time reaction. The findings might not be generalizable to other metal complexes. Still, caution must be taken when a metal complex is used under harsh conditions of oxidation or reduction reactions, especially where the related metal, metal oxide/salt, is active for the corresponding reaction. Such metal complexes are extensively used as catalysts for different reduction and oxidation reactions. The approach based on various techniques is expected to inspire a thorough revision of other key molecular catalyst systems to reveal the true active species. This is critical for advances in the field of catalysis to set the guidelines for informed molecular catalyst designs.

## Methods

All reagents and solvents were obtained from commercial sources and used without further purification unless otherwise stated. SBA-15 (Sigma-Aldrich) and manganese(III) 5,10,15,20-tetra(4-pyridyl)-21H,23H-porphine chloride tetrakis(methochloride) (**1**) (Sigma-Aldrich) were purchased. Iodosylbenzene (PhIO) was prepared by the hydrolysis^48^ of iodosylbenzenediacetate (Sigma-Aldrich, 98%). Cyclohexene (Merck) was purified with a short-activated silica column, CHCl_3_ (≥ 99%, Merck), CH_3_CN (99.8%, Merck), and DI water (18-20 MΩ•cm^-1^ at 27 °C) were used.

### Immobilization of 1 on mesoporous SBA was done according to the literature procedure^49^

Manganese(III) 5,10,15,20-tetra(4-pyridyl)-21H,23H-porphine chloride tetrakis (methochloride) (**1**) (12 µmol as 10^−3 ^mol L^−1^), and SBA (1.00 g) in DI water were stirred at 25 °C for 6 h. The suspension was filtered by centrifugation, and the amount of immobilized **1** on SBA was determined by UV–Vis spectroscopy^50^. The solid was washed with deionized water, methanol, ethanol, and chloroform, respectively, until the absence of **1** in the washings was monitored by UV–Vis spectroscopy. The collected solid was dried at 80 °C for 24 h.

### Immobilization of MnAc on silica SBA

It was synthesized by electrostatic interaction between the positive charges of the manganese(II) ions and the Si-OH groups present on the surface. An aqueous solution of manganese(II) acetate (1.0 mM) and SBA-15 (1.00 g) were placed under magnetic stirring at 25 °C. After 6 h, the suspension was filtrated, washed, and dried at 50 °C.

### The oxidation reaction of cyclohexene by PhIO catalyzed by 1/SBA

The oxidation reaction was performed in 5 mL vials sealed with a septum at room temperature, in the dark, under air, and magnetic stirring for 90 min. Cyclohexene (100 µL), SBA-**1** (0.2 µmol of **1**), PhIO (0.43 mg (2.0 μmol) or 4.30 mg (20.0 μmol)) were added in 500 µL of the solvent mixture CH_3_CN: CHCl_3_ (1:1, v/v) with a molar ratio of SBA-**1**:PhIO:Cyclohexene of 1:10:5000 or 1:100:5000. The reaction was monitored by gas chromatography (GC) using bromobenzene as an internal standard.

Control reactions with MnAc/SBA: PhIO, or with PhIO (without **1** or SBA), and or SBA (without **1**) were also carried out under the same conditions, which described for the 1 h/SBA: PhIO reaction.

### Characterization

SEM was carried out using an LEO 1430VP microscope. HRTEM and TEM were carried out using an FEI Tecnai G^2^ F20 transmission electron microscope, TF20 (200 kV). The X-ray powder diffraction patterns were recorded with a Bruker D8 Advance (Germany) diffractometer (CuK_α_ radiation). X-ray photoelectron spectroscopy (XPS) measurements were performed by an X-Ray BesTec XPS system (Germany) with an Al K_α_ X-ray source (hυ = 1,486.6 eV). EDX analysis/mapping was carried out with the scanning electron microscope CamScan 4DV (CamScan UK). FTIR spectra of the materials prepared as KBr pellets were recorded on a Bruker vector 22 between 400 and 4000 cm^-1^. The electron paramagnetic resonance (EPR) measurements were carried out using a Bruker EMX spectrometer working at a fixed frequency of 9.38 GHz (X-band). A 100 kHz magnetic field modulation and phase-sensitive detection were used to record the derivative of the absorbed microwave power. The amplitude of absorption was normalized by the mass of samples to compare different samples.

### XAS measurements

XAS measurements (EXAFS and XANES) at the manganese K-edge for **1**, SBA-**1**, and SBA-**1** after the reaction were performed at the KMC-3 beamline at the BESSY II synchrotron facility (Helmholtz-Zentrum Berlin, Germany). The measurements were performed at 20 K using a liquid-helium-cooled cryostat (Oxford-Danfysik) in the top-up mode of the BESSY II storage ring at 250 mA ring current. The angle between the sample surface and the incoming X-ray beam was ≈ 45°. The fluorescence-detected X-ray absorption spectra at the K-edge were collected using a 13-element Ge detector (Ultra-LEGe detector, Canberra GmbH) installed perpendicular to the incident X-ray beam.

## Supplementary information

Supplementary Information PDF

Description of Additional Supplementary File

Supplementary Data 1

## Data Availability

All data generated or analyzed during this study are included in this published article [and its supplementary information files].
