# Supplementary material for: Role of decomposition products in the oxidation of cyclohexene using a manganese(III) complex
Source: Commun Chem. 2023 May 17;6:94. doi: 10.1038/s42004-023-00881-x (PMC10192294; doi:10.1038/s42004-023-00881-x)
Supplement: Supplementary file 1 — Supplementary Information PDF [file 42004_2023_881_MOESM1_ESM.pdf]

Supplementary Information

Role of decomposition products in the oxidation of cyclohexene using a manganese(III) complex

Zahra Zand,[a] Younes Mousazade,[a] Ryan Lacdao Arevalo,[b] Robabeh Bagheri,[c] Mohammad Reza Mohammadi,[d] Rahman Bikas,[e] Petko Chernev,[f,g] Pavlo Aleshkevych,[h] Matthias Vandichel,[b] Zhenlun Song,[c] Holger Dau[f] and Mohammad Mahdi Najafpour*[a]

[a]Department of Chemistry, Institute for Advanced Studies in Basic Sciences (IASBS), Zanjan, 45137-66731, Iran

[b]Department of Chemical Sciences and Bernal Institute, University of Limerick, Limerick, V94 T9PX, Ireland

[c]Key Laboratory of Marine Materials and Related Technologies, Zhejiang Key Laboratory of Marine Materials and Protective Technologies, Ningbo Institute of Materials Technology and Engineering, Chinese Academy of Sciences, Ningbo 315201, China

[d]University of Sistan and Baluchestan, Department of Physics, Zahedan, 98167-45845, Iran

[e]Department of Chemistry, Faculty of Science, Imam Khomeini International University, 34148-96818 Qazvin, Iran

[f]Fachbereich Physik, Freie Universit-t Berlin, Arnimallee 14, 14195 Berlin, Germany

[g]Department of Chemistry, Ångström Laboratory, Uppsala University, Box 523, Uppsala 751 20, Sweden

[h]Institute of Physics, Polish Academy of Sciences (PAN), Al. Lotnikow 32/46, PL-02-668 Warsaw, Poland

*Corresponding Author:

mmnajafpour@iasbs.ac.ir

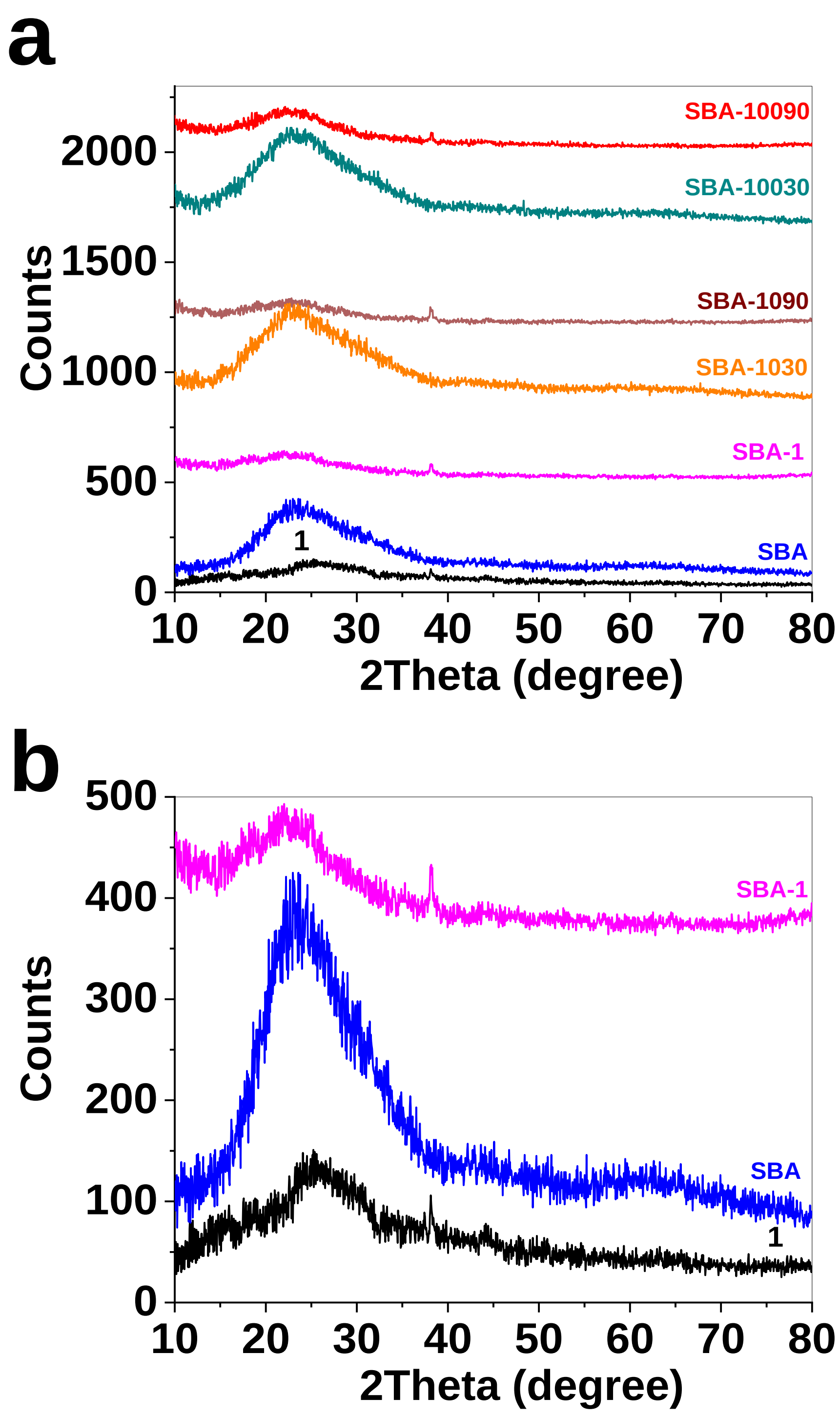

Supplementary Figure 1. XRD patterns. XRD patterns for SBA, **1**, SBA-**1**, SBA-**1030**, SBA-**1090**, SBA-**10030**, and SBA-**10090** (a,b).

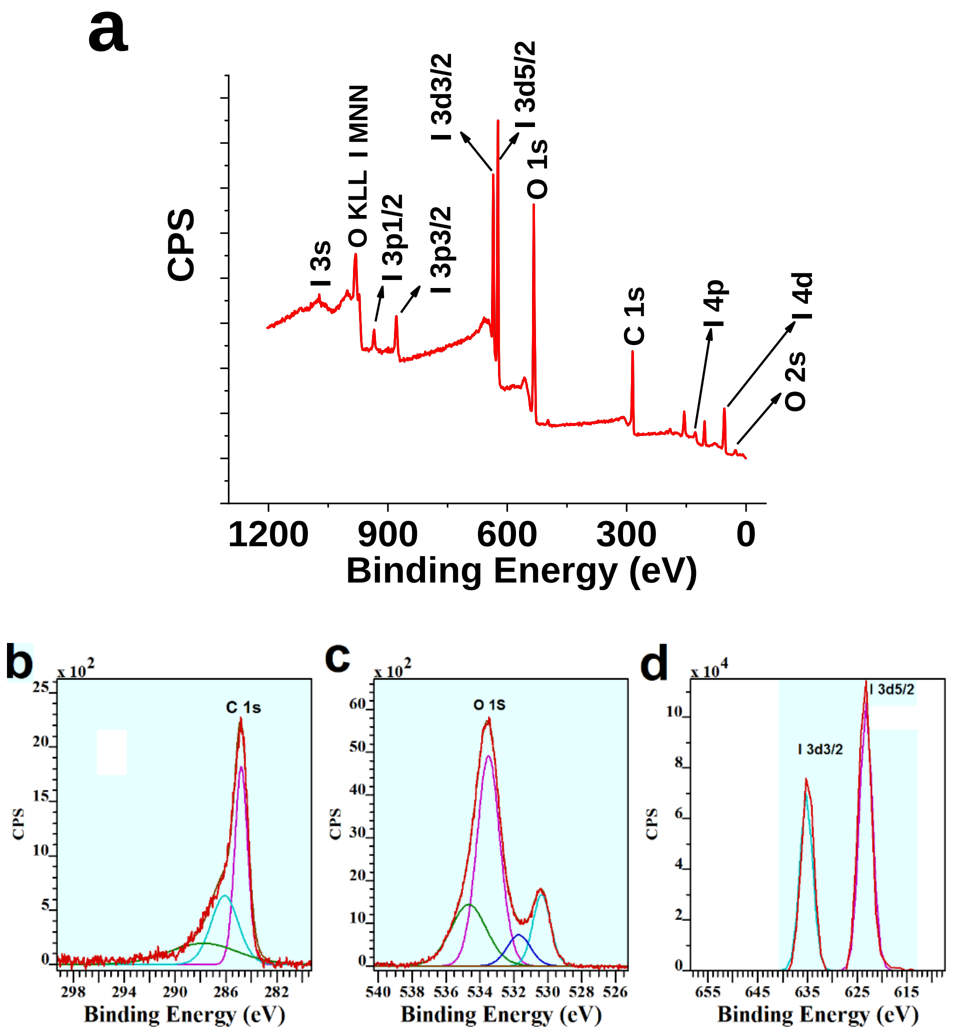

Supplementary Figure 2. XPS spectra. XPS spectrum of SBA-**10030** (a). XPS spectra of SBA-**10030** at C (b)**,** O (**c**), and I (d).

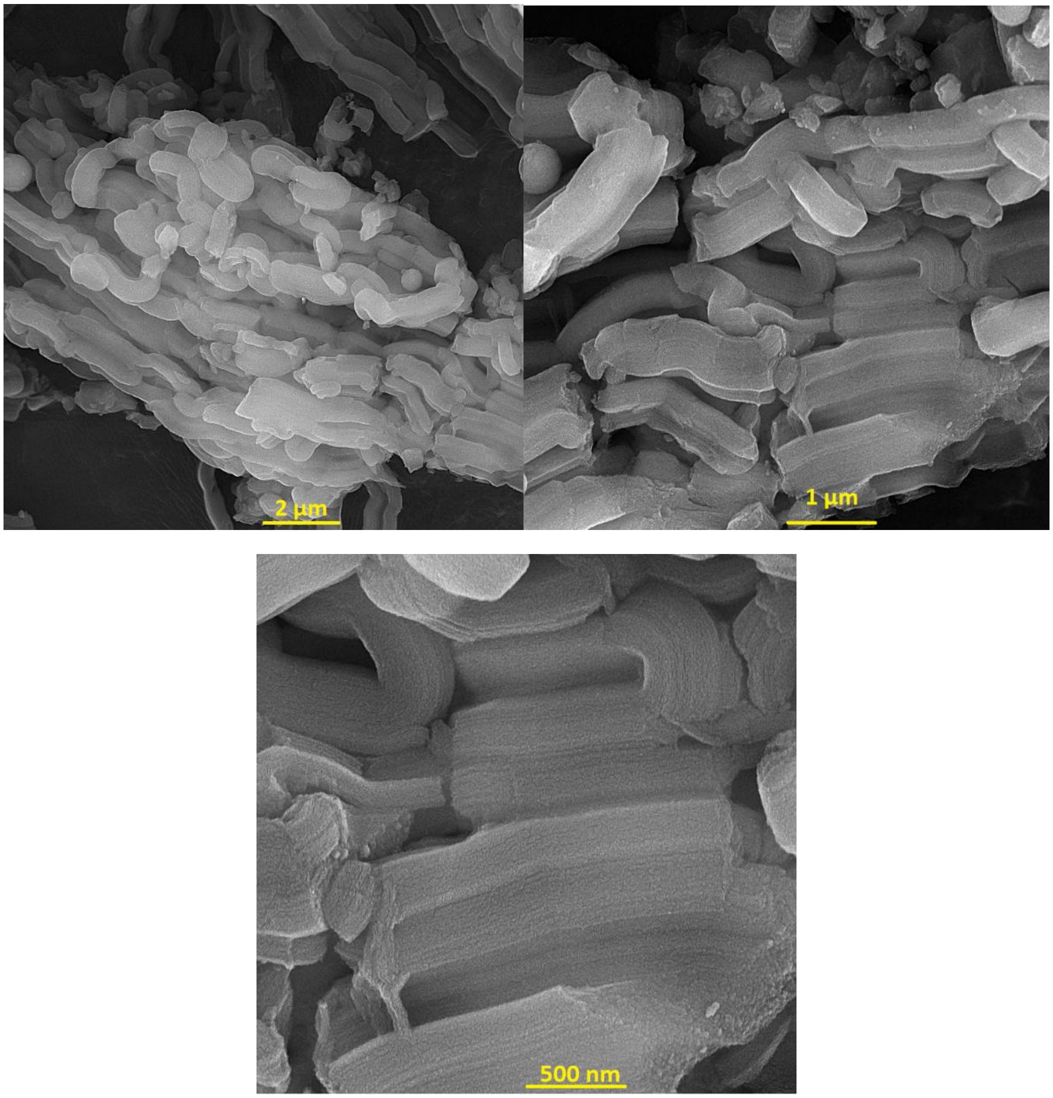

Supplementary Figure 3. SEM images. SEM images of SBA at different magnifications.

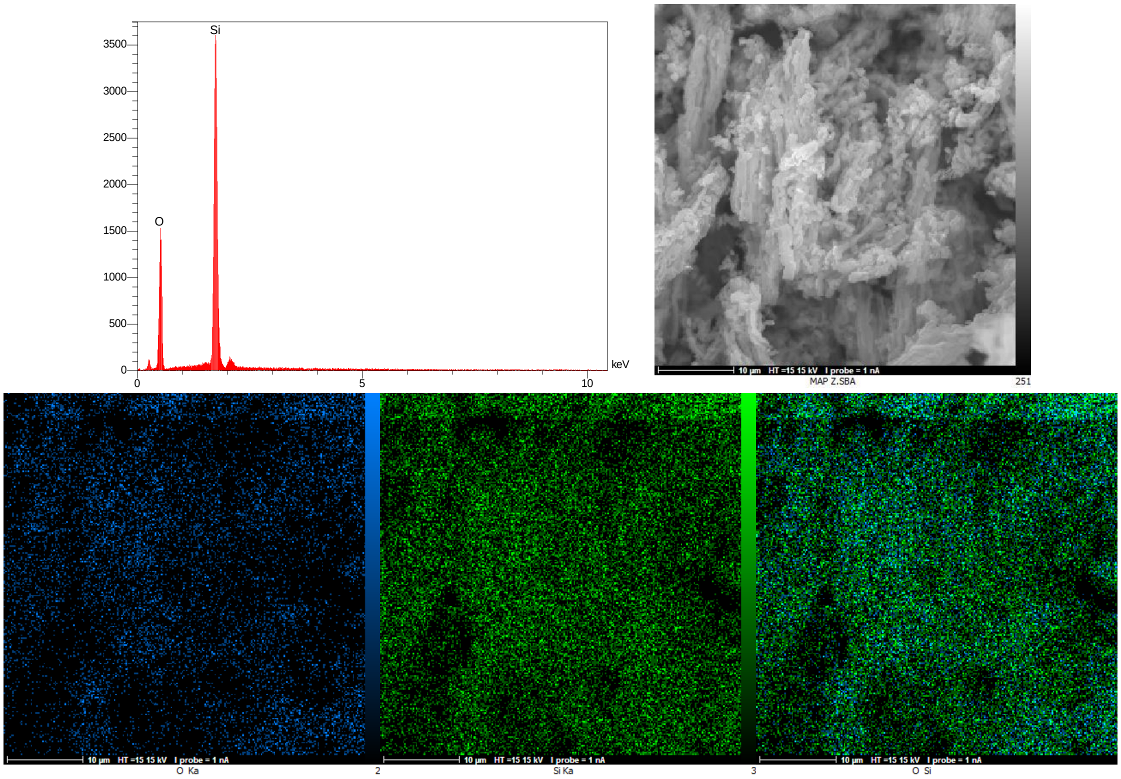

Supplementary Figure 4. SEM-EDX Mapping and spectrum. SEM-EDX Mapping and spectrum of SBA.

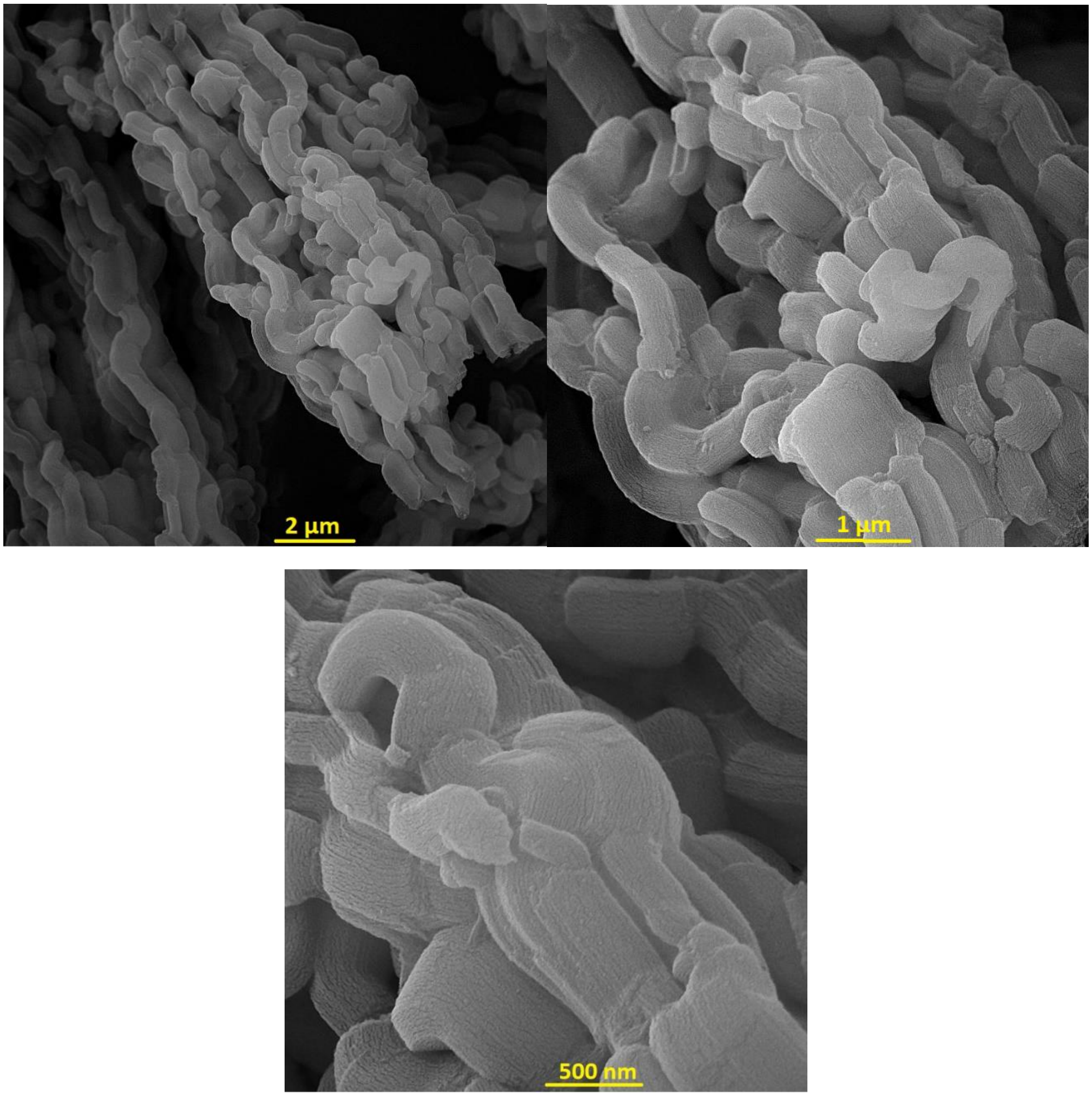

Supplementary Figure 5. SEM images. SEM images of SBA-**1** at different magnifications.

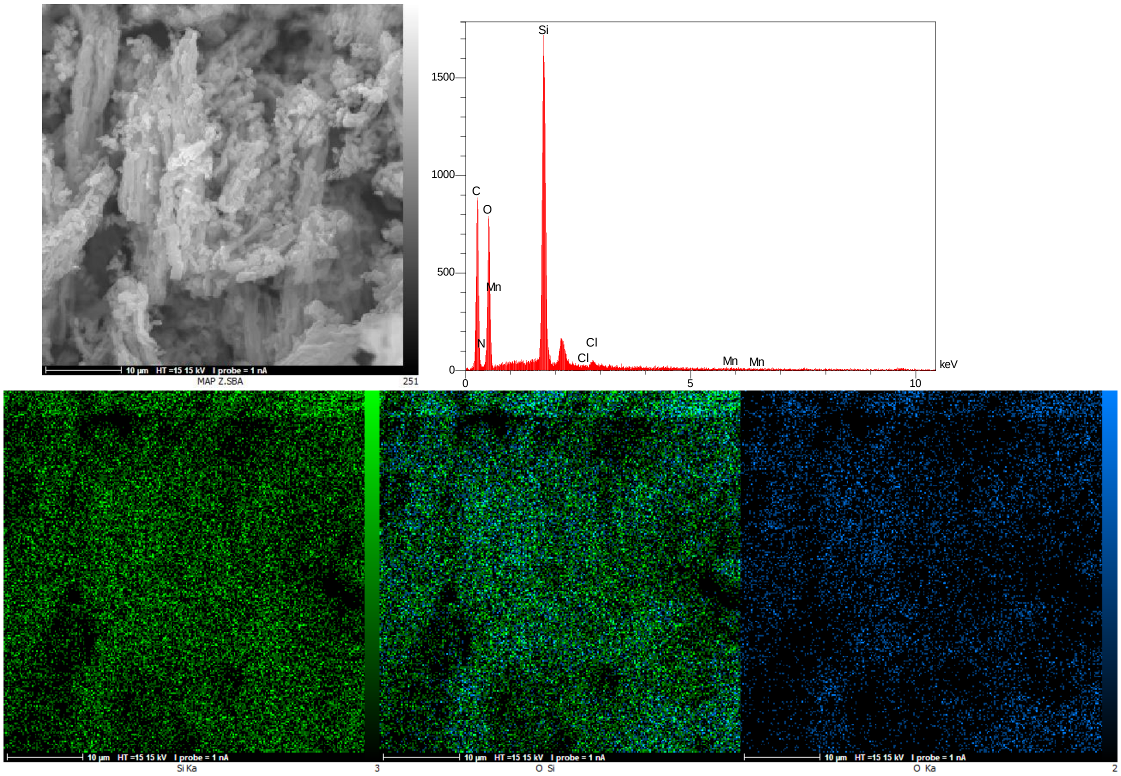

Supplementary Figure 6. SEM-EDX Mapping and spectrum. SEM-EDX Mapping and spectrum of SBA-**1**.

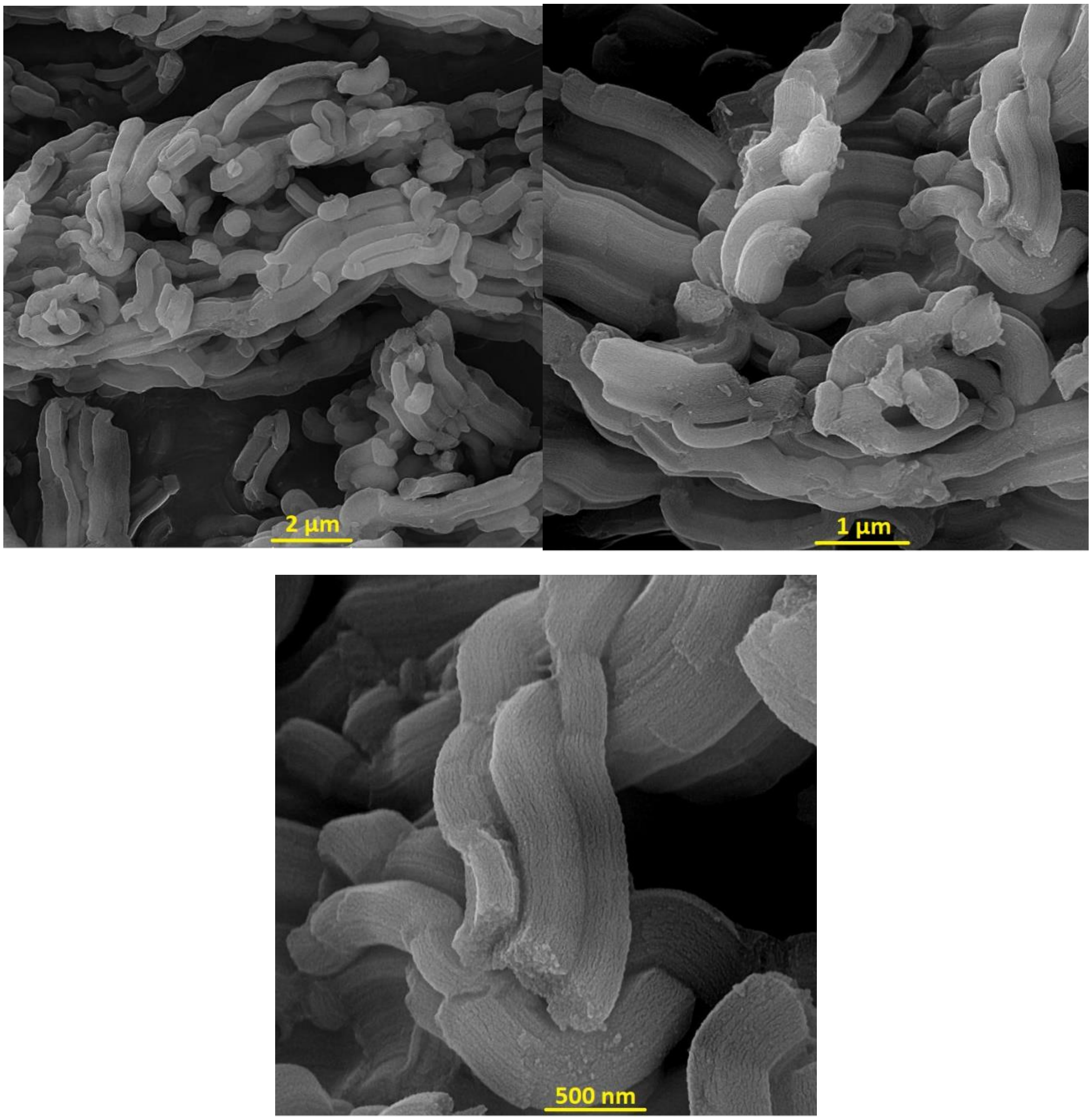

Supplementary Figure 7. SEM images. SEM images of SBA-**1030** at different magnifications.

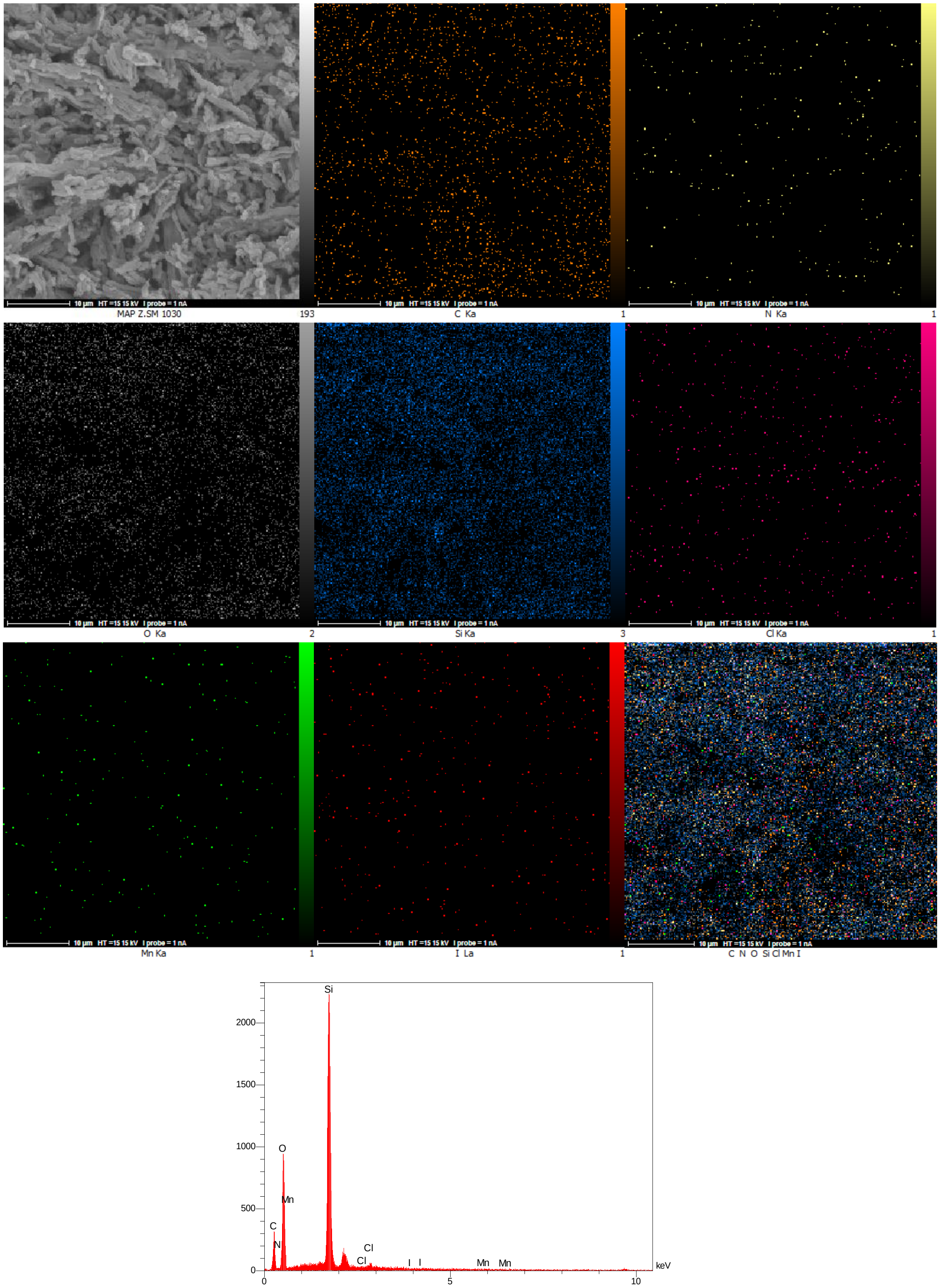

Supplementary Figure 8. SEM-EDX Mapping and spectrum. SEM-EDX Mapping and spectrum of SBA-**1030**.

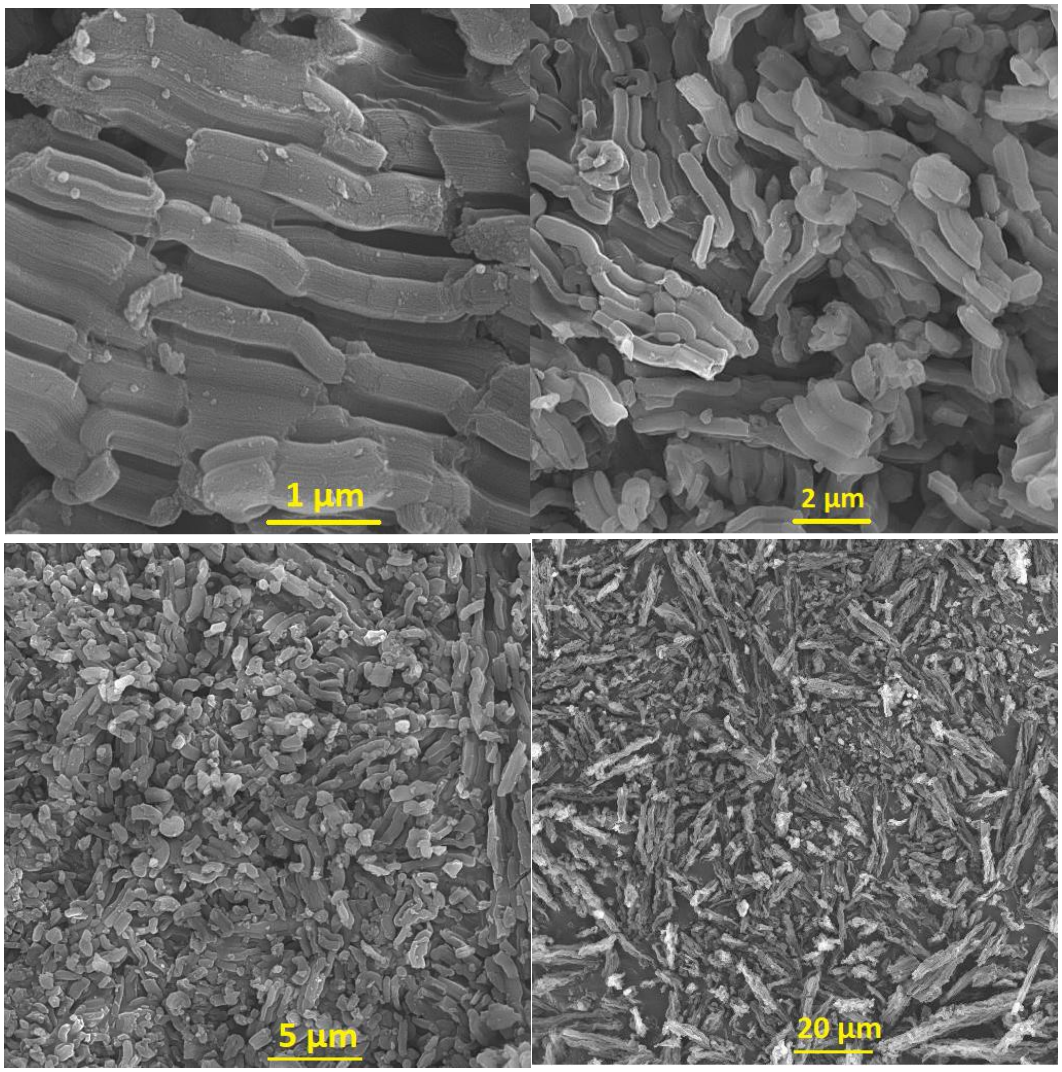

Supplementary Figure 9. SEM images. SEM images of SBA-**1090** at different magnifications.

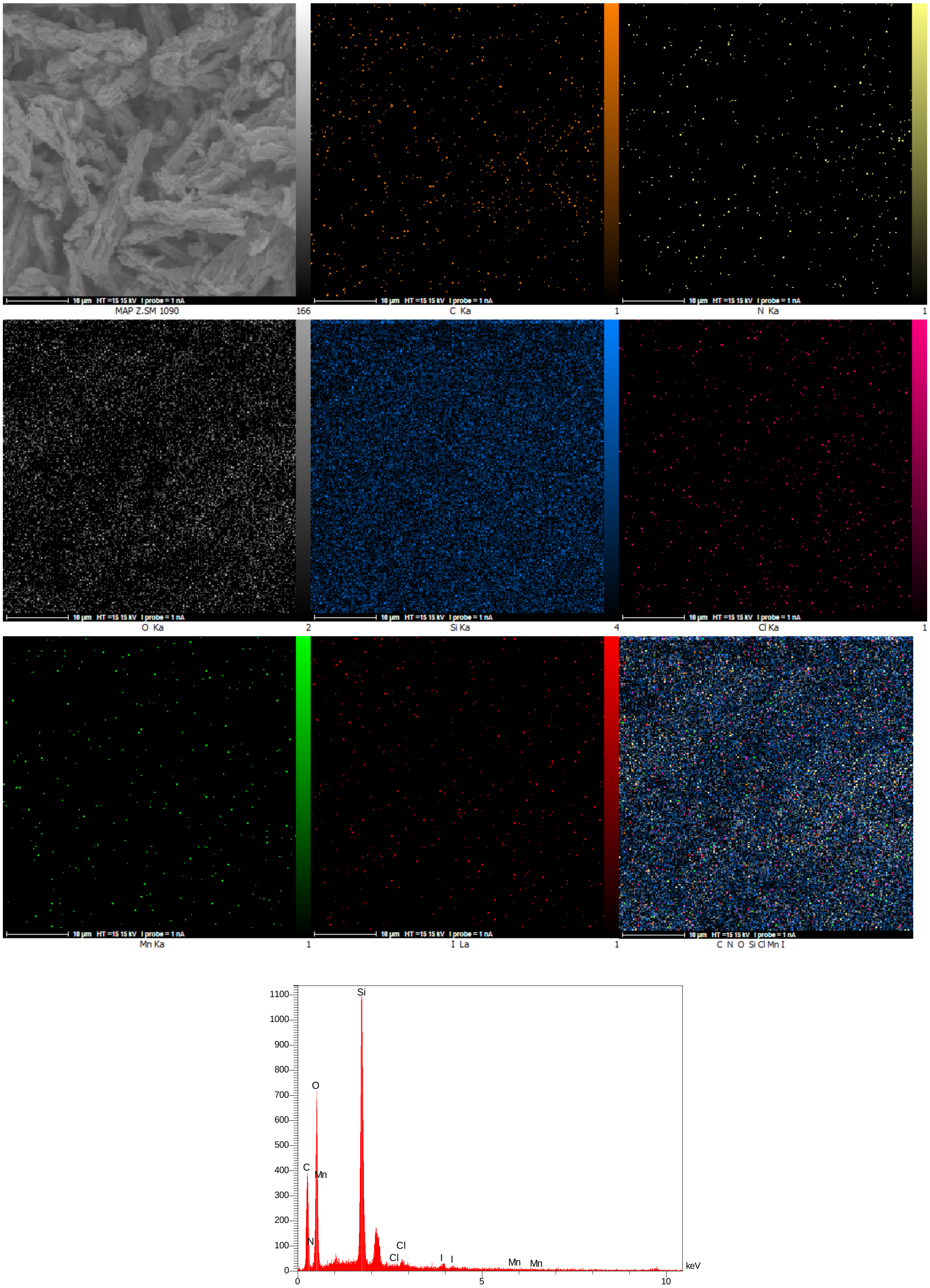

Supplementary Figure 10. SEM-EDX Mapping and spectrum. SEM-EDX Mapping and spectrum of SBA-**1090**.

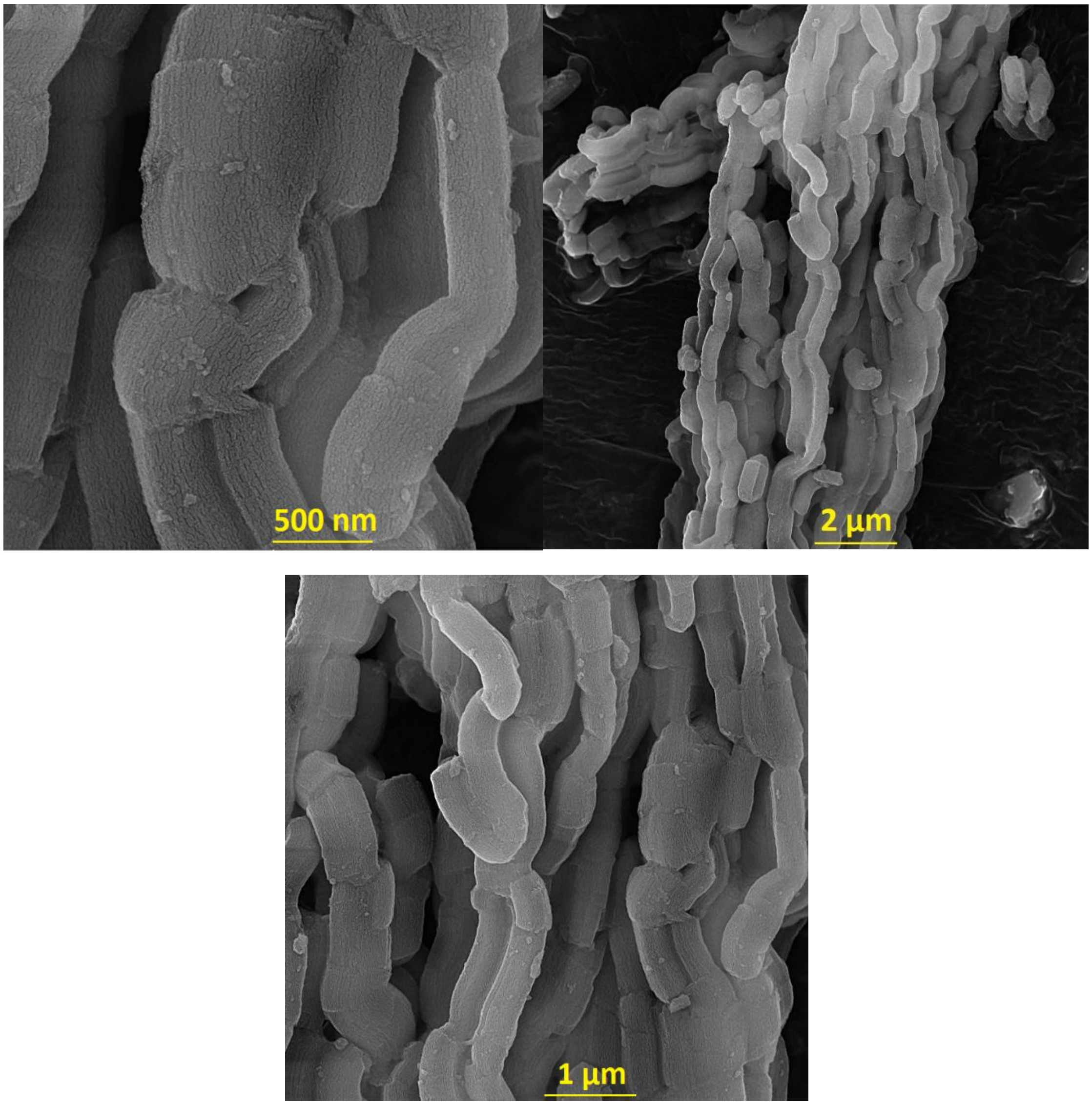

Supplementary Figure 11. SEM images. SEM images of SBA-**10030** at different magnifications.

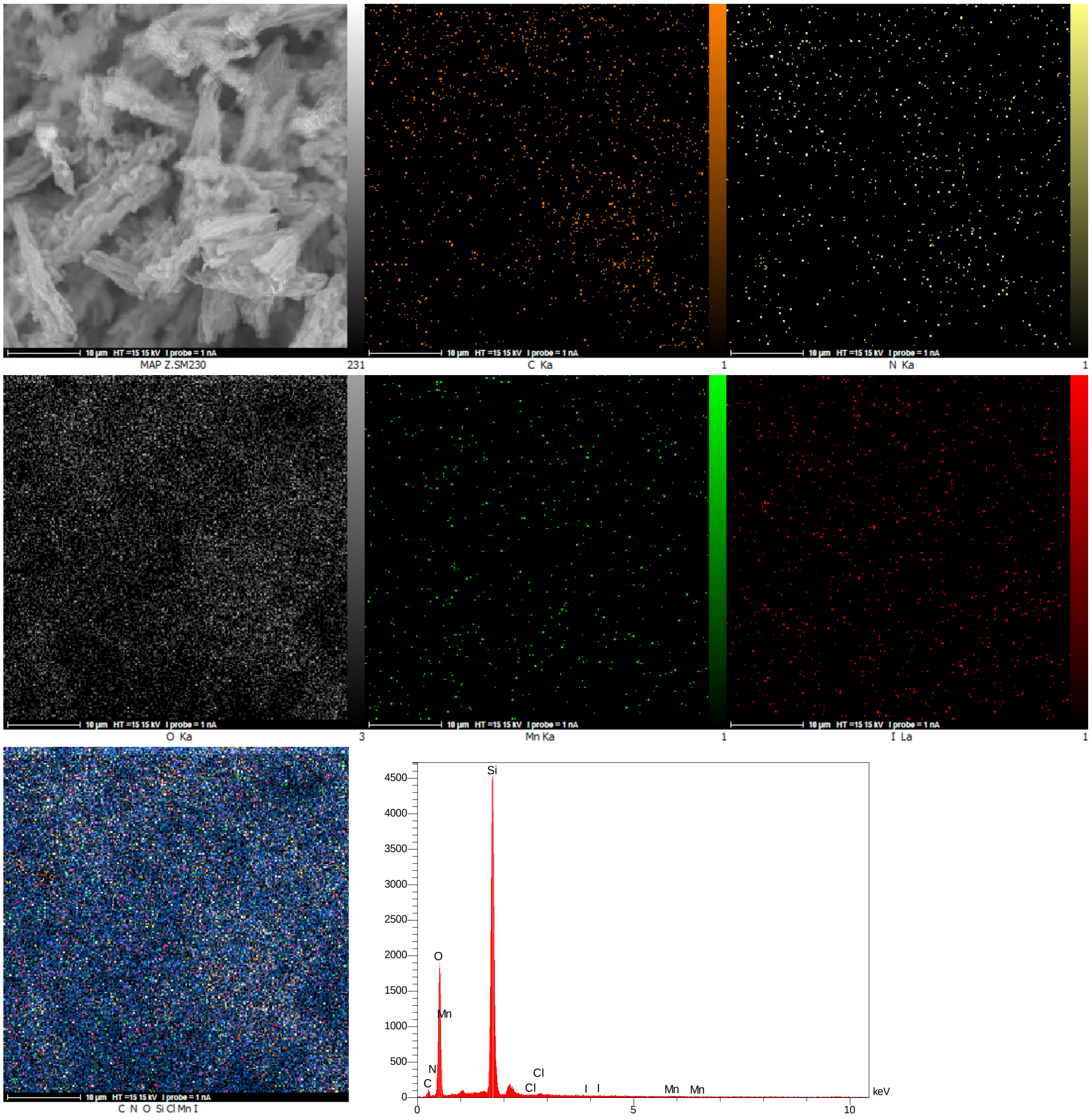

Supplementary Figure 12. SEM-EDX Mapping and spectrum. SEM-EDX Mapping and spectrum of SBA-**10030**.

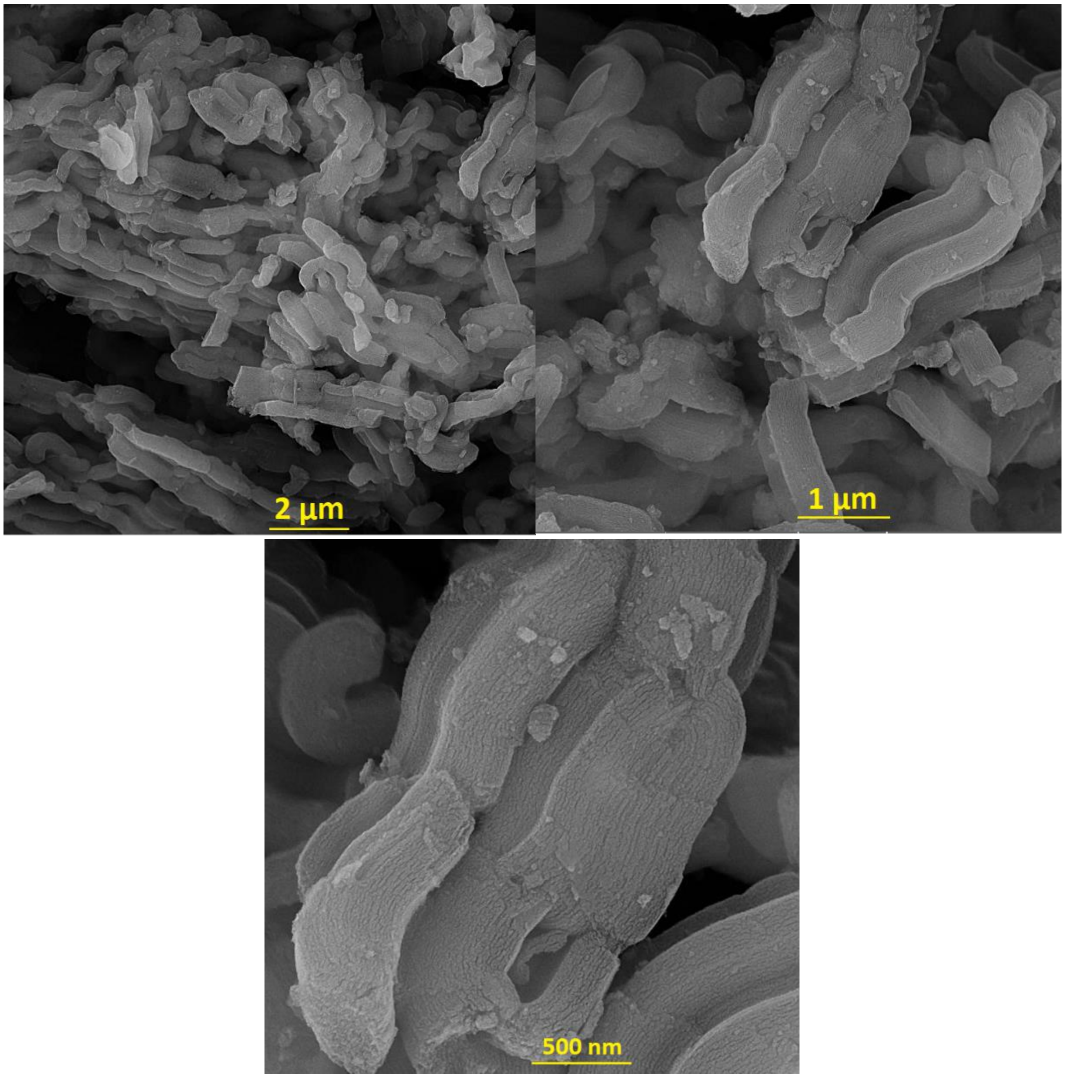

Supplementary Figure 13. SEM images. SEM images of SBA-**10090** at different magnifications.

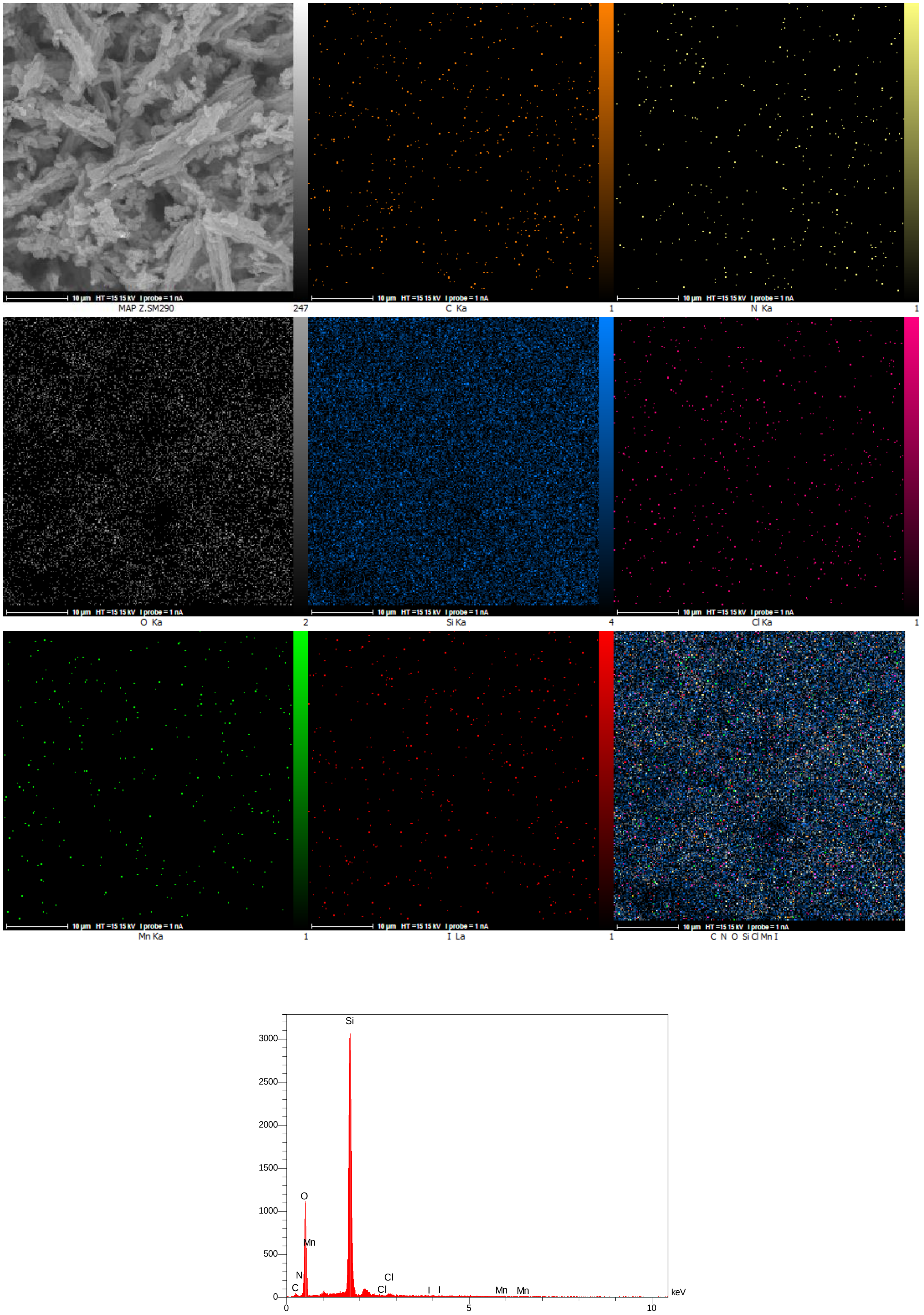

Supplementary Figure 14. SEM-EDX Mapping and spectrum. SEM-EDX Mapping of SBA-**10090**.

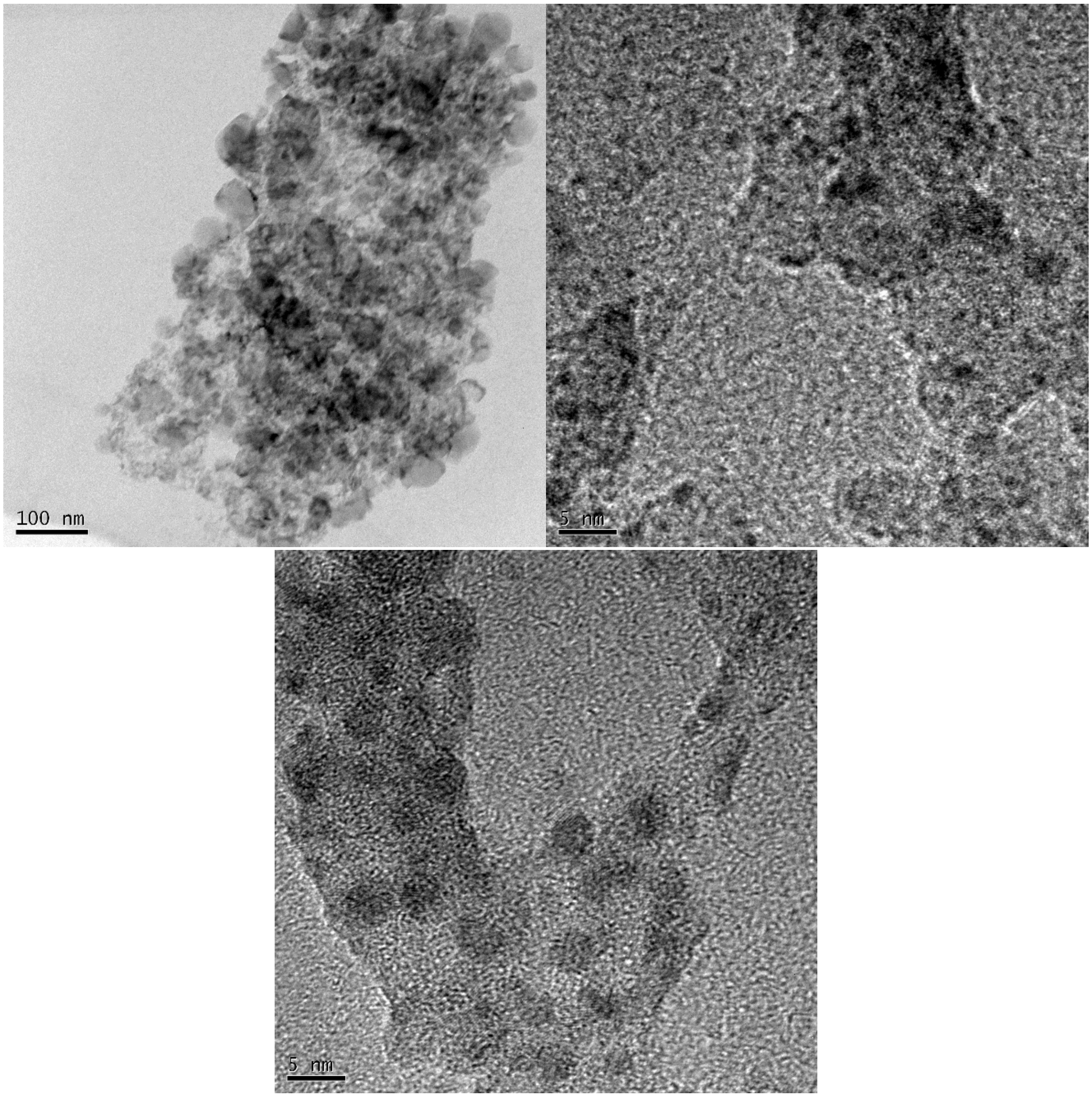

Supplementary Figure 15. (HR)TEM images. TEM images of SBA-**1** at different magnifications.

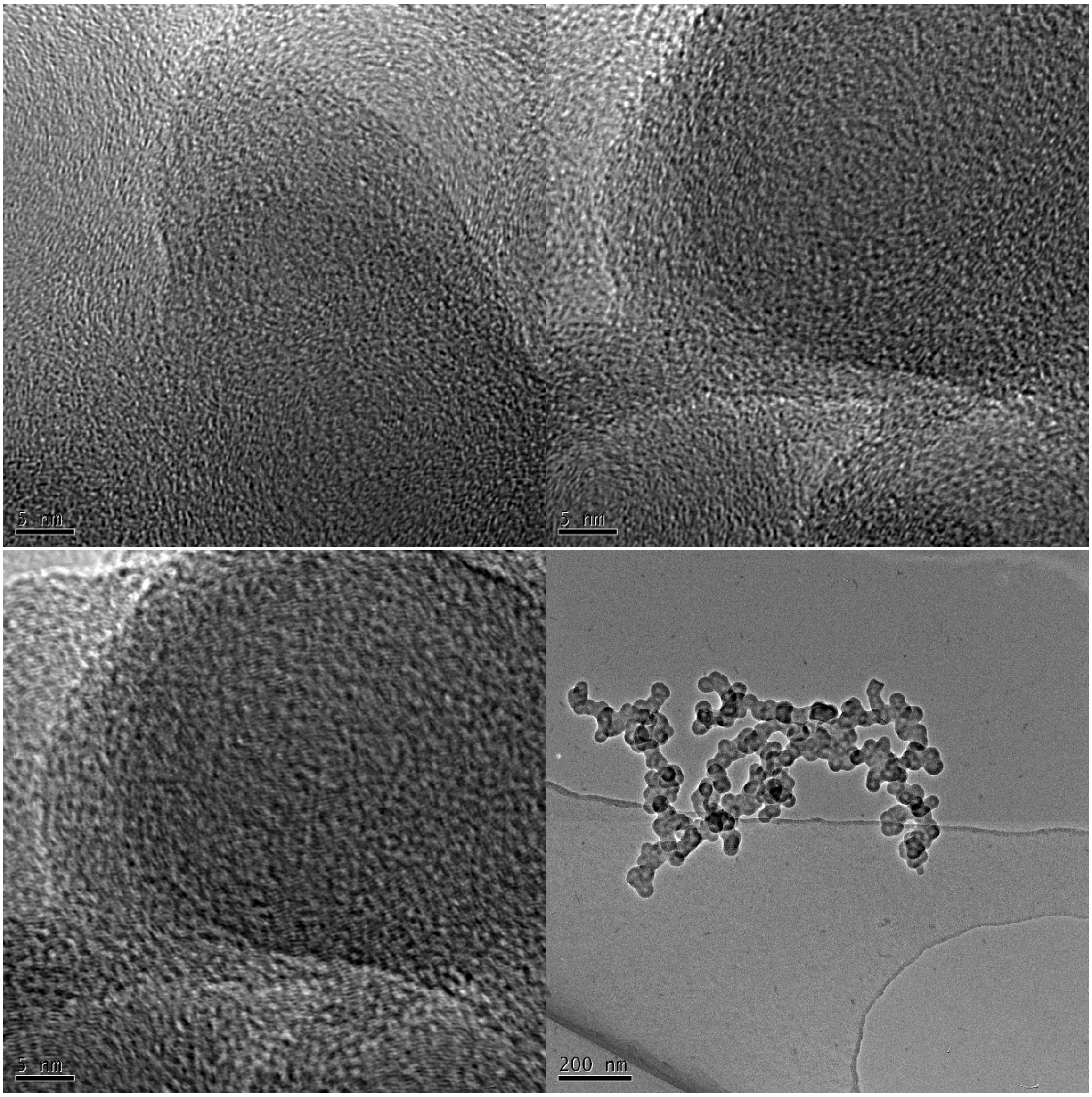

Supplementary Figure 16. (HR)TEM images. (HR)TEM images of SBA-**1030** at different magnifications.

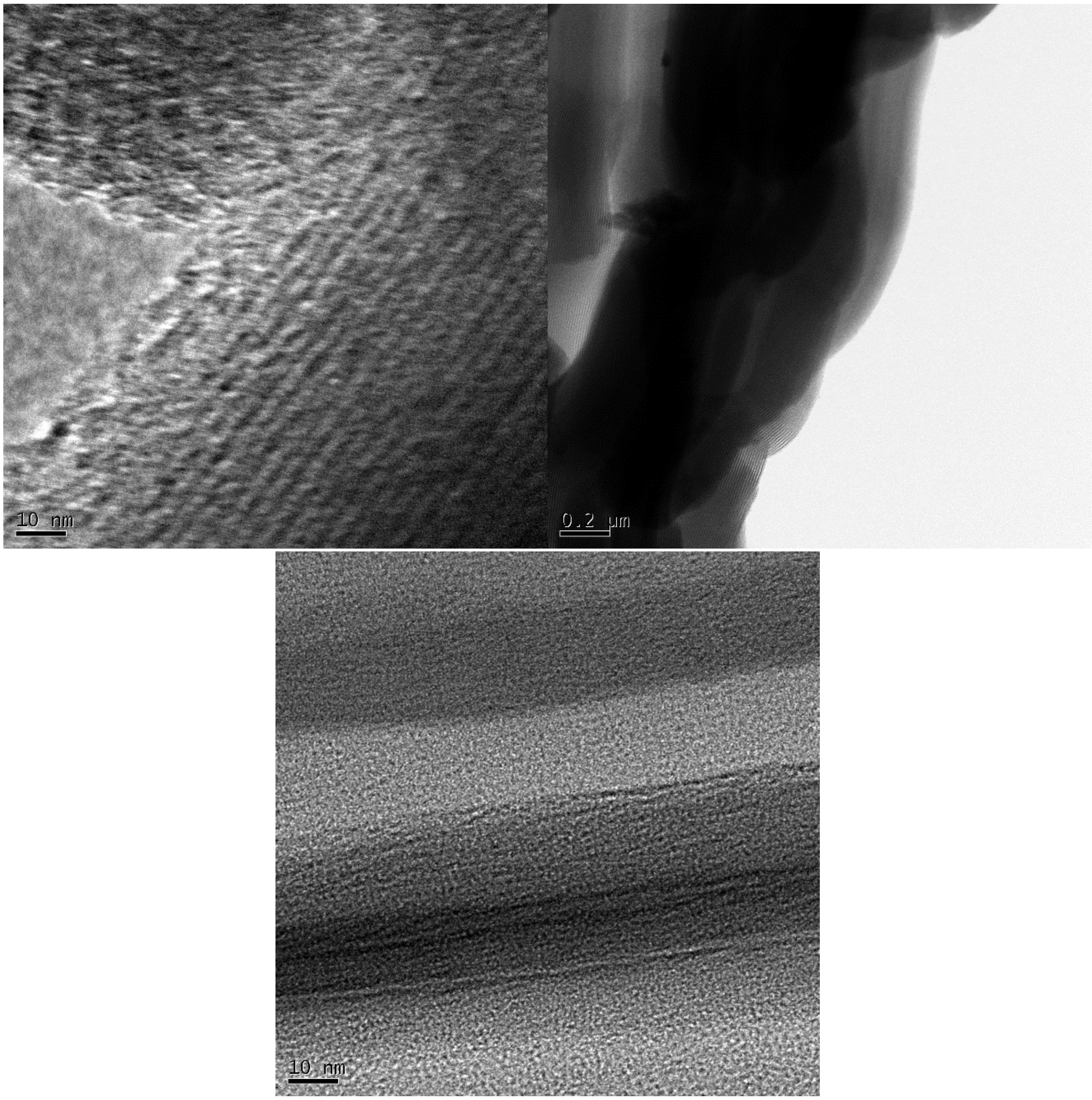

Supplementary Figure 17. (HR)TEM images. (HR)TEM images of SBA-**1090** at different magnifications.

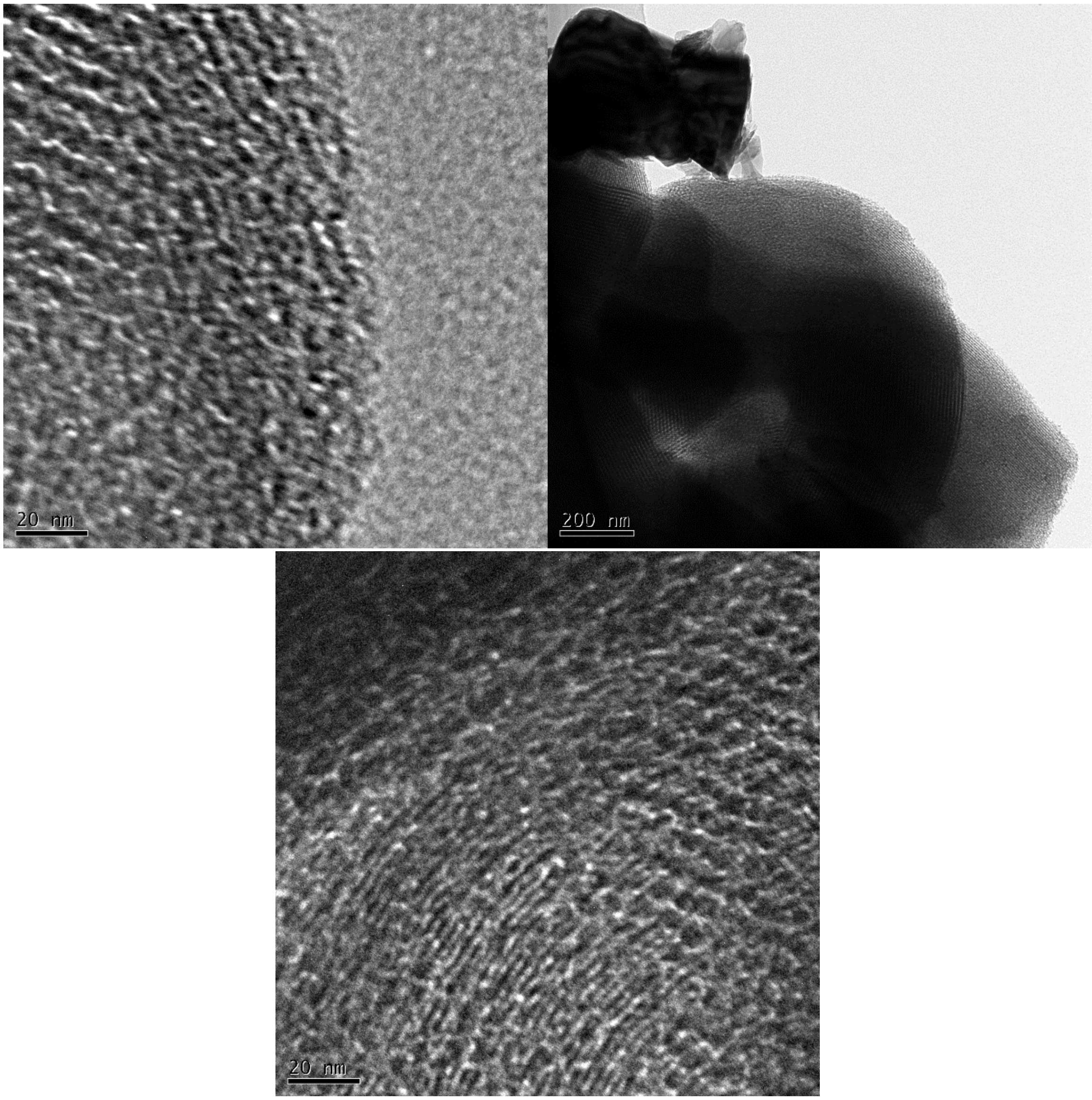

Supplementary Figure 18. (HR)TEM images. (HR)TEM images of SBA-**10030** at different magnifications.

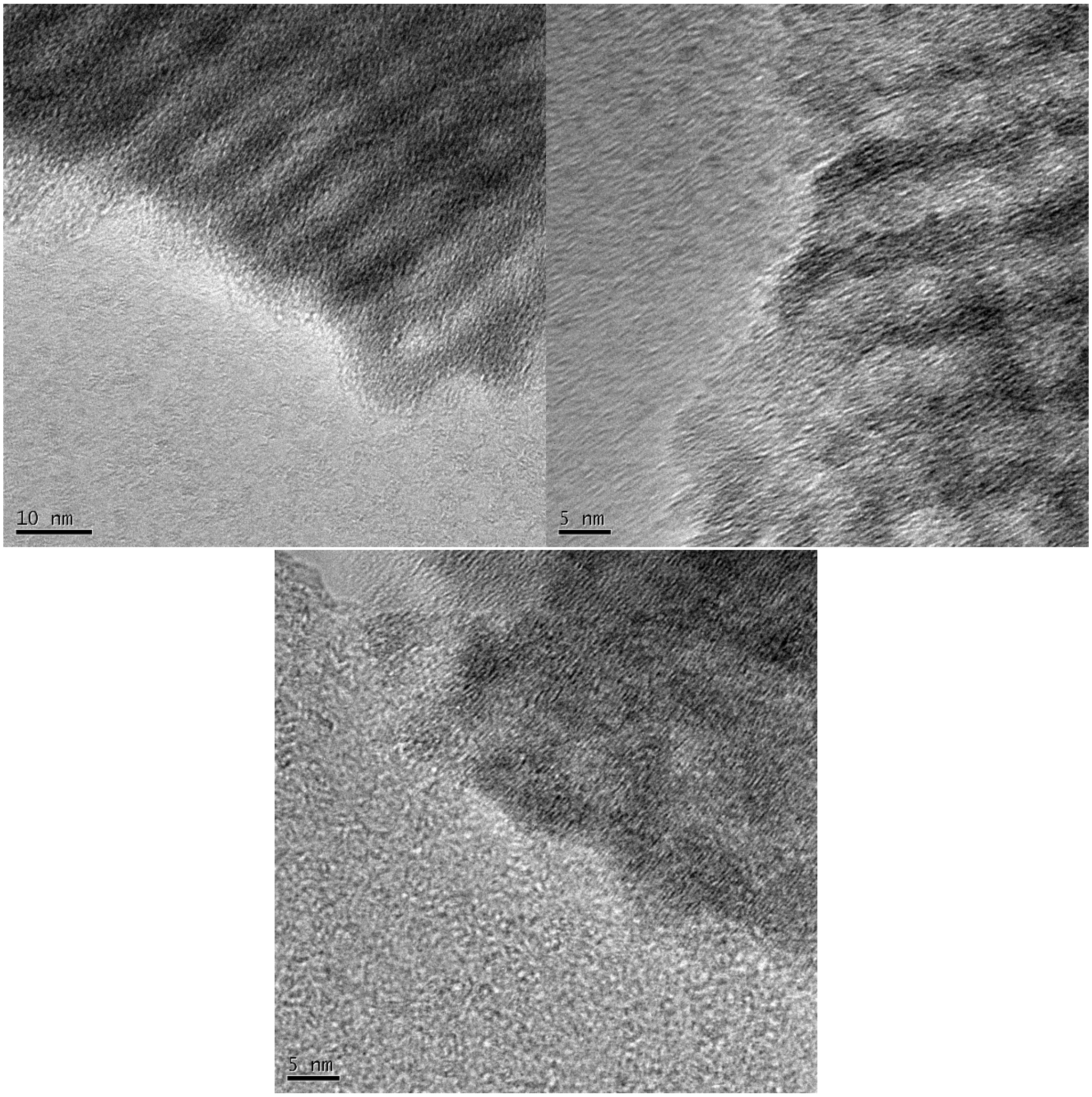

Supplementary Figure 19. (HR)TEM images. (HR)TEM images of SBA-**10090** at different magnifications.

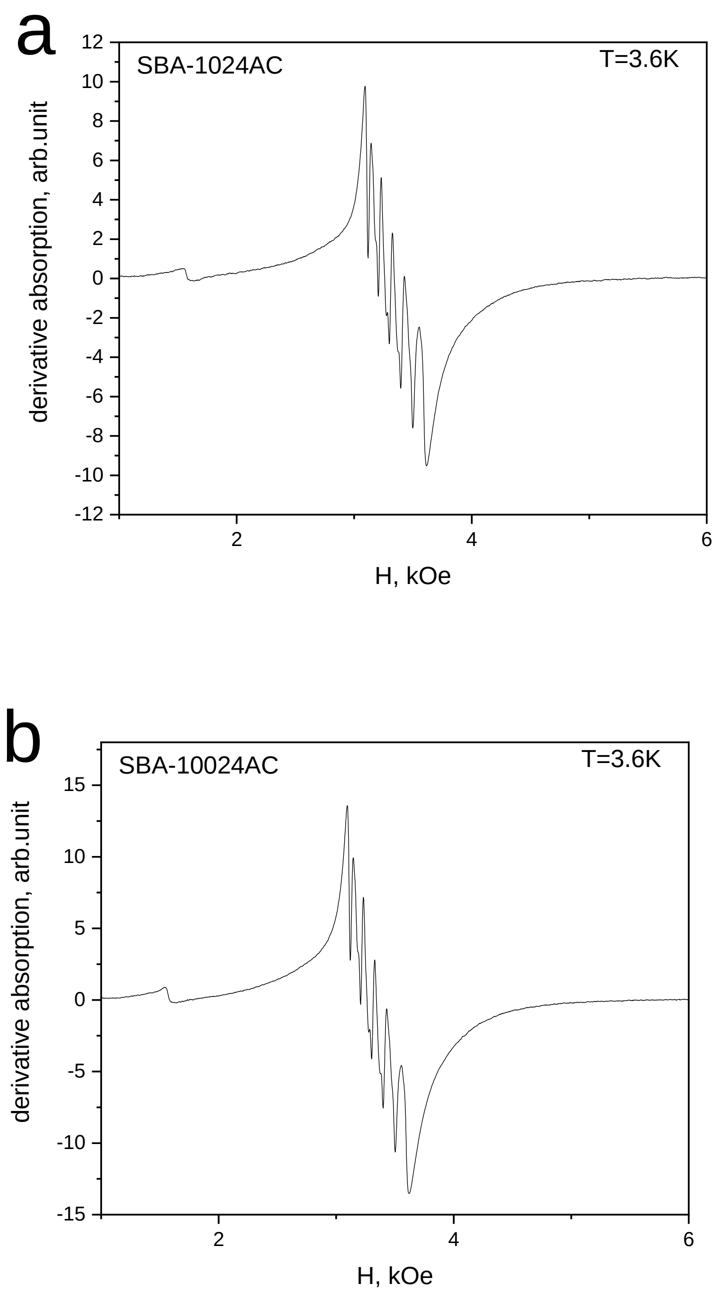

Supplementary Figure 20. EPR spectra. EPR spectra for SBA-**1024AC** (a) and SBA-**10024AC** recorded at liquid helium temperature.

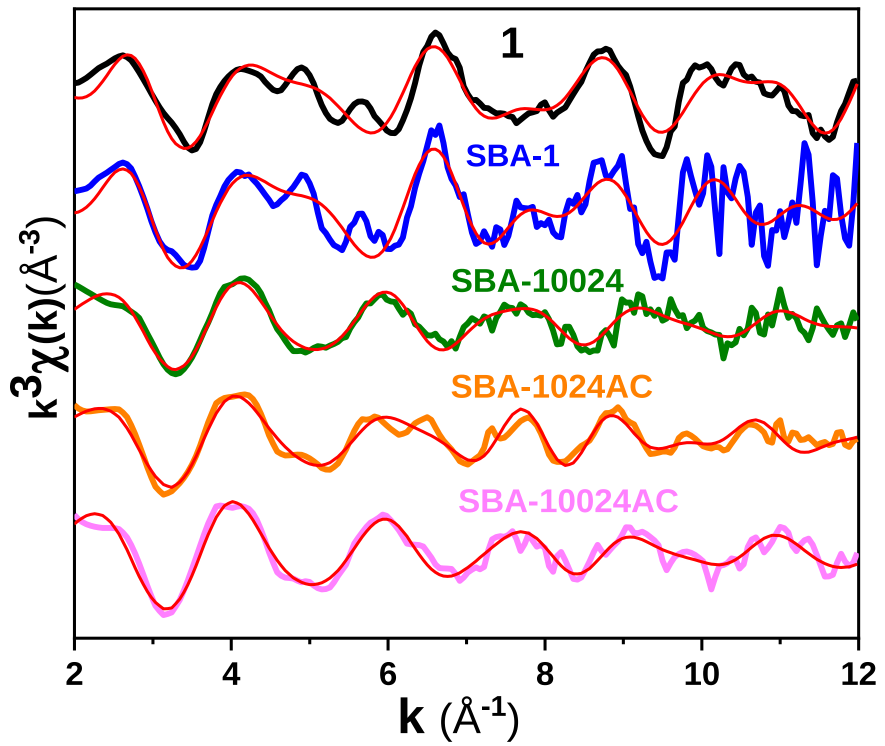

Supplementary Figure 21. EXAFS spectra. K^3-weighted χ(k) of EXAFS spectra of **1** (black), SBA-**1** (blue), SBA-**10024** (green)**,** SBA-**1024AC** (orange)and SBA-**10024AC** (pink). The thick lines show experimental data, and the thin red lines show simulations. The fit parameters for the simulations are given in Table **1**. Phase shift not corrected.

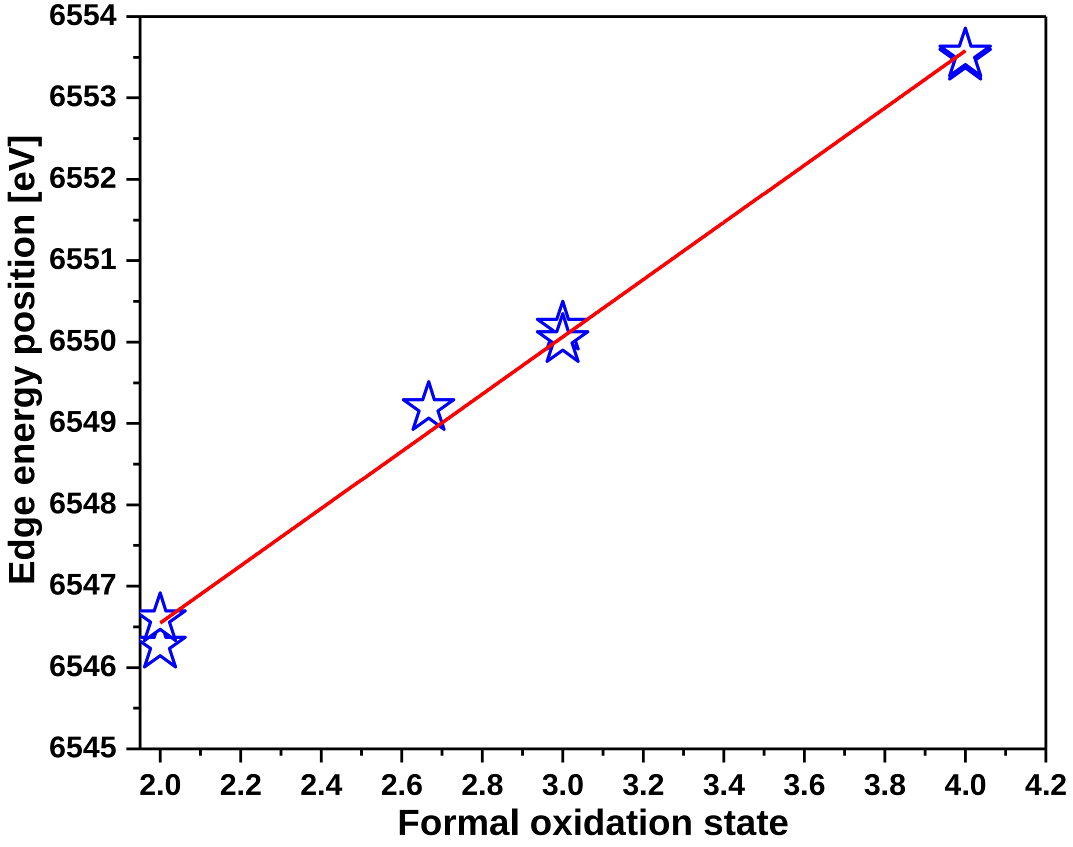

Supplementary Figure 22. Mn *K*-edge XANES. Mn *K*-edge XANES of $Mn^{II}O$, $Mn^{II}CO_3$, Mn_3O_4, $Mn^{III}{}_2O_3$, α-$Mn^{III}{}_2O_3$, $Mn^{IV}O_2$, and β-$Mn^{IV}O_2$ were measured as references.

Supplementary Table S1 The oxidation of cyclohexene in the presence of PhI(AC)$_2$ under different conditions.

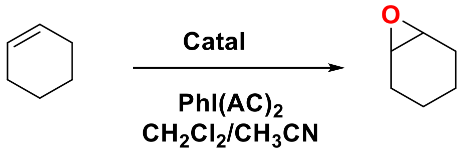

Entry	Catalyst	Oxidant/ Catalyst	Time	Yield
1	SBA-**1**	10/1	24 h	20%
2	SBA/MnAc	10/1	24 h	10%
3	SBA-**1**	100/1	24 h	15%
4	SBA-**1**	100/1	6 h	10%
5	SBA/MnAc	100/1	24 h	9%
6	Reused SBA-**1**	100/1	24h	8%
7	-	10/0	24h	%2>
8	SBA	10/1	24h	%2>

Supplementary Table 2 The oxidation of cyclohexene in the presence of TBA-OX under different conditions.

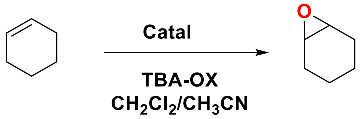

Entry	Catalyst	Oxidant/ Catalyst	Time	Conversion
1	SBA-**1**	10/1	24 h	0
2	SBA/MnAc	10/1	24 h	0
3	SBA-**1**	100/1	24 h	0
4	SBA/MnAc	100/1	24 h	0
5 [b]	Reused SBA-**1**	100/1	24h	0
6	-	10/0	24h	0
7	SBA	10/1	24h	0

[a]GC yield.

[b] The Reused SBA-**1** catalyst was recovered from the reaction of entry 3.

Supplementary Table 3 The oxidation of cyclohexene in the presence of MCPBA under different conditions.

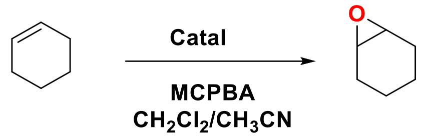

Entry	Catalyst	Oxidant/ Catalyst	Time	conversion
1	SBA-**1**	10/1	24 h	0
2	SBA/MnAc	10/1	24 h	0
3	SBA-**1**	100/1	24 h	0
4	SBA/MnAc	100/1	24 h	0
5 [b]	Reused SBA-**1**	100/1	24h	0
6	-	10/0	24h	0
7	SBA	10/1	24h	0

[a]GC yield.

[b] The Reused SBA-**1** catalyst was recovered from the reaction of entry 3.
