# Supplementary material for: Role of decomposition products in the oxidation of cyclohexene using a manganese(III) complex
Source: Commun Chem. 2023 May 17;6:94. doi: 10.1038/s42004-023-00881-x (PMC10192294; doi:10.1038/s42004-023-00881-x)
Supplement: Supplementary file 2 — Description of Additional Supplementary File [file 42004_2023_881_MOESM2_ESM.pdf]

Description of Additional Supplementary Files

File name: Supplementary Data 1

Description: The xyz coordinates of atoms for Points A-E
